# Left–right asymmetric cell intercalation drives directional collective cell movement in epithelial morphogenesis

**DOI:** 10.1038/ncomms10074

**Published:** 2015-12-10

**Authors:** Katsuhiko Sato, Tetsuya Hiraiwa, Emi Maekawa, Ayako Isomura, Tatsuo Shibata, Erina Kuranaga

**Affiliations:** 1 Laboratory for Physical Biology, RIKEN Center for Developmental Biology, Kobe 650-0047, Japan; 2 Laboratory for Histogenetic Dynamics, RIKEN Center for Developmental Biology, Kobe 650-0047, Japan; 3 Laboratory of Molecular Cell Biology and Development, Graduate School of Biostudies, Kyoto University, Kyoto 606-8501, Japan; 4 Laboratory for Tissue Development Dynamics, Graduate School of Biological Sciences, Nara Institute of Science and Technology, Nara 630-0192, Japan; 5 Laboratory for Histogenetic Dynamics, Graduate School of Life Sciences, Tohoku University, Sendai 980-8577, Japan

## Abstract

Morphogenetic epithelial movement occurs during embryogenesis and drives complex tissue formation. However, how epithelial cells coordinate their unidirectional movement while maintaining epithelial integrity is unclear. Here we propose a novel mechanism for collective epithelial cell movement based on *Drosophila* genitalia rotation, in which epithelial tissue rotates clockwise around the genitalia. We found that this cell movement occurs autonomously and requires myosin II. The moving cells exhibit repeated left–right-biased junction remodelling, while maintaining adhesion with their neighbours, in association with a polarized myosin II distribution. Reducing *myosinID*, known to cause counter-clockwise epithelial-tissue movement, reverses the myosin II distribution. Numerical simulations revealed that a left–right asymmetry in cell intercalation is sufficient to induce unidirectional cellular movement. The cellular movement direction is also associated with planar cell-shape chirality. These findings support a model in which left–right asymmetric cell intercalation within an epithelial sheet drives collective cellular movement in the same direction.

## Abstract (web summary)

Coordinated epithelial movement during embryogenesis drives complex tissue formation, but how this movement is coordinated to maintain epithelial integrity is not clear. Here the authors show that left-right asymmetry in cell intercalation drives clockwise rotation of epithelia in *Drosophila* genital development.

## Graphical abstract

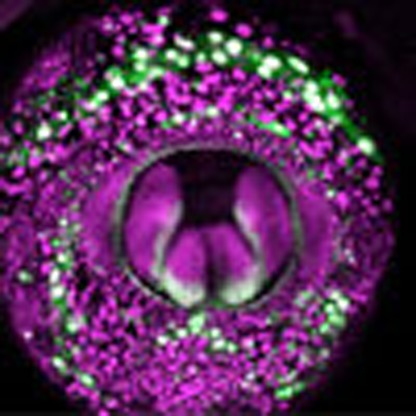

Epithelial morphogenesis is achieved by individual cells acting collectively within epithelial sheets, while maintaining the tissue's integrity. To deform an epithelial sheet, the cells have to relocate themselves collectively or synchronously. The mechanisms underlying such collective cellular movements have been extensively researched by studying the gastrulation of various model organisms. The fundamental cellular and molecular machineries identified in gastrulation also operate in organogenesis, wound healing and cancer metastasis. One of the best-studied examples of collective cell movement in epithelial morphogenesis is convergent extension, which causes tissue elongation through the rearrangement of polarized cells via cell intercalation^1^. Convergent extension drives axis elongation in the dorsal mesoderm of *Xenopus*^2^ and zebrafish^3^, the kidney tubule of *Xenopus*^4^ and the germ band of *Drosophila*^5^. Mechanical tension on cell junctions appears to play a role in these morphological processes^5^,^6^. For example, in *Drosophila* germ band elongation, cell junctions perpendicular to the anterior–posterior (AP) axis accumulate high levels of non-muscle myosin II (Myo-II), which increases the strength of the junctional tension, accompanied by a decrease in junctional length, whereas cell junctions parallel to the AP axis have low levels of Myo-II and tend to expand^5^. This process is mediated by the polarized remodelling of the adherens junctions, protein complexes at cell–cell junctions that contain actomyosin cables and adhesion molecules such as E-cadherin^7^,^8^,^9^,^10^.

Recent studies have illuminated the roles of the collective movement of cohesive cell clusters in epithelial cell sheets in the formation of complex tissues^11^. The type of collective cell movement that relies on the leading edge of a moving cell cluster that senses extrinsic guidance cues has been intensively investigated, and its mechanisms are well-understood^11^,^12^. However, there are also examples of cell clusters lacking a leading edge that undergo collective movement while maintaining their epithelial characteristics, such as in tracheal invagination^11^, mammary gland sprouting^11^ and eyelid closure in mice^13^, and in egg chamber rotation in *Drosophila*^14^,^15^,^16^,^17^.

Another example of collective cell movement without a leading edge is the clockwise rotation of *Drosophila* genitalia. *Drosophila* male terminalia undergo a 360° clockwise rotation starting about 24 h after puparium formation (APF) and concluding 36–38 h APF; this rotation induces dextral spermiduct looping around the hindgut (Fig. 1a). During metamorphosis, the genital imaginal disc, which includes three embryonic segments (A8 tergite, A9 genitalia and A10 analia), is partially everted, exposing its apical surface and adopting a circular shape while remaining attached to the A7 epidermis (Fig. 1b)^18^. Genitalia rotation is reported to be controlled by the combined half rotations of two A8 domains, the anterior (A8 anterior: A8a) and posterior (A8 posterior: A8p) compartments of A8 (Fig. 1b). A portion of the cells in A8p, along with A9 and A10 initially rotates 180°, whereas A8a continues to rotate the remaining 180°, which causes the genitalia to rotate the entire 360° (Fig. 1c,c' and Supplementary Movie 1)^19^,^20^. The conserved type ID unconventional myosin 31DF gene (*myosinID*: *myoID*) contributes to the clockwise direction of genitalia rotation^21^ and is transcriptionally controlled by the Hox gene, *AbdB*^22^; however, the mechanism by which the rotation is achieved is unknown.

Here we investigate the *Drosophila* genitalia rotation process, especially that of A8a, and propose a new scenario for collective cell movement that maintains epithelial integrity. In the model, left–right (LR) asymmetrically polarized Myo-II accumulation is induced within the apical plane of epithelial cells, followed by polarized junction remodelling and cell intercalation. Using live imaging analysis, we found that genitalia rotation involves the clockwise movement of the surrounding epithelial tissue and that this process can be recapitulated *ex vivo*. The moving cells undergo intercalation, whereas remaining attached to neighbouring cells. Most of the remodelled cell boundaries form right oblique angles with the AP axis and are associated with LR biased Myo-II accumulation. The polarized Myo-II distribution is reversed by disrupting *myoID*. Numerical simulation shows that the LR asymmetric contractility and junction remodelling are sufficient to induce directional cell movement in virtual two-dimensional tissues. The individual epithelial cells surrounding the genitalia adopts the LR asymmetry in shape, which is related to the directional movement, both *in vivo* and *in silico*. These findings provide new mechanistic insight into the directional collective cell movement that induces the LR asymmetric morphogenesis of epithelial tissue.

## Results

### Myo-II-related epithelial movement leads to organ rotation

The deformation of developing epithelial tissues along the body axis involves an epithelial cell rearrangement process that is induced by planar polarized junctional contractility elicited by Myo-II, and junctional shortening caused by the endocytosis of E-cadherin^5^,^7^,^23^,^24^. Thus, we first examined Myo-II's contribution to the genitalia rotation. For this analysis, the A8p and A8a rotations were marked by fluorescent proteins expressed by the *engrailed (en)-GAL4* and the *Abdominal B (AbdB)-GAL4*^*LDN*^ drivers, respectively^19^. We found that *en-GAL4*- and *AbdB-GAL4*^*LDN*^-driven expressions did not overlap in the A8 segment (Supplementary Fig. 1a,b). As *en-GAL4* is known to drive expression in the posterior component of each segment^25^, this result indicates that *AbdB-GAL4*^*LDN*^ drives expression only in A8a.

**Figure 1:**
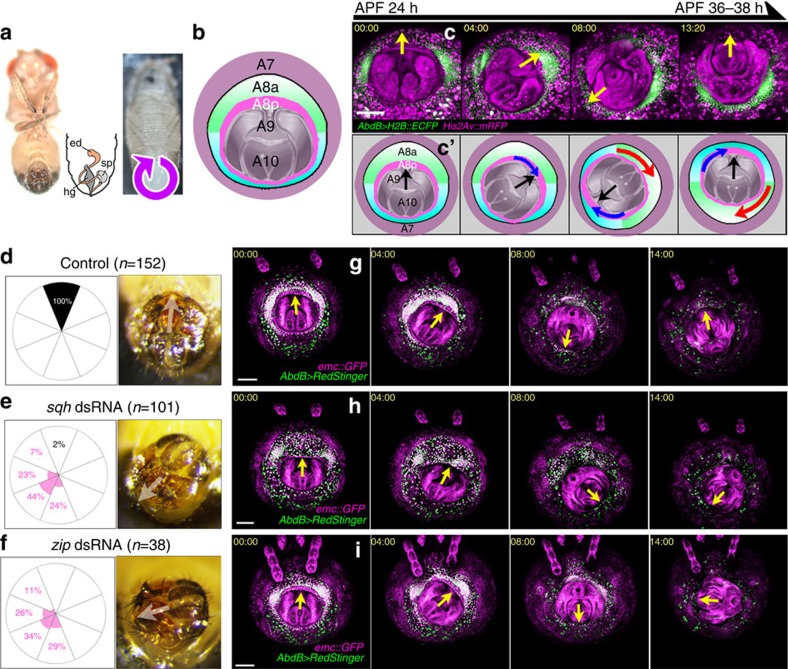
Genitalia rotation requires Myo-II. (**a**) Adult male (left) and pupae (right). Schematic of the adult male abdomen (middle), showing the looping of the ejaculatory duct (ed) around the hindgut (hg) resulting from a 360° clockwise rotation. sp, sperm pump. (**b**) Schematic of male genitalia at 24 h APF. The posterior part of the A8 tergite (magenta) surrounds the innermost parts (grey), including the genitalia (A9) and analia (A10). The anterior part of A8 (green: ventral part, light blue: dorsal part) is surrounded by A7 epidermis (mauve). (**c**) Time-lapse series of genitalia rotation. Ventral is at the top. Green: nuclei in anterior A8 (A8a) cells, visualized by enhanced cyan fluorescent protein (ECFP) expressed by the *AbdB-GAL4*^*LDN*^ driver. Magenta: all nuclei, visualized by *His2Av::mRFP*. Yellow (or black in **c**') arrows indicate the direction from the analia to the external genitalia. **c** is schematically drawn in **c**'. Blue and red arrows indicate the movement of A8p and A8a, respectively. Scale bar, 100 μm. (**d**–**f**) The external genitalia pointed to the ventral side in all control flies (**d**), whereas most of the flies expressing *sqh* (**e**) dsRNA and *zip* (**f**) dsRNA with the *AbdB-GAL4*^*LDN*^ driver showed orientation defect. White arrows indicate the direction from the analia to the external genitalia. Rose diagrams indicate the frequency of the external genitalia position in adult male flies. (**g**–**i**) Time-lapse series of genitalia rotation in control (**g**), *sqh* dsRNA (**h**) and *zip* dsRNA-expressing flies (**i**). Green: nuclei in A8a, visualized by RedStinger with the *AbdB-GAL4*^*LDN*^ driver. Magenta: *emc::GFP*, which labels almost all nuclei. Yellow arrows indicate the direction from the analia to the external genitalia. Ventral is at the top. Scale bar, 100 μm. Fly genotypes: *His2Av::mRFP/+; AbdB-GAL4 UAS-H2B::ECFP/+* (**c**), *UAS-Dcr2/+; AbdB-GAL4 UAS-RedStinger/+* (**d**), *UAS-Dcr2/+; AbdB-GAL4 UAS-RedStinger/UAS-sqh dsRNA* (**e**), *UAS-Dcr2/+; AbdB-GAL4 UAS-RedStinger/UAS-zip dsRNA* (**f**), *UAS-Dcr2/+; emc::GFP AbdB-GAL4 UAS-RedStinger/+* (**g**), *UAS-Dcr2/+; emc::GFP AbdB-GAL4 UAS-RedStinger/UAS-sqh dsRNA* (**h**)*, UAS-Dcr2/+; emc::GFP AbdB-GAL4 UAS-RedStinger/UAS-zip dsRNA* (**i**).

First, we reduced the Myo-II level in A8a, by expressing the double-strand RNA (dsRNA) of *spaghetti squash* (*sqh*, which encodes the Myo-II regulatory light chain) or *zipper* (*zip*, which encodes the myosin heavy chain), using *AbdB-GAL4*^*LDN*^. These flies showed impaired genitalia rotation and orientation of the adult terminalia (Fig. 1e,f,h,i); control flies showed full rotation (Fig. 1d,g). In contrast, *sqh* or *zip* knockdown in the A8p using *en-GAL4* did not affect the orientation of adult male terminalia (Supplementary Fig. 1c–e). These findings indicated that the expression of Myo-II specifically in A8a is critical for genitalia rotation.

These data suggested that A8a might rotate using a type of cellular movement equivalent to that seen in epithelial tissue deformation. To examine this possibility, we first analysed the cellular status of the A8 tergite during rotation. The male genital imaginal disc is derived from the endoderm and forms an epithelial monolayer at the larval stage. Staining for *Drosophila* E-cadherin (*D*E-Cad) and Discs Large (Dlg, a septate junction marker) at 29 h APF (during rotation) revealed that the cells adhered to each other even as rotation progressed (Supplementary Figs 2 and 3). Cross-sectional images revealed that the A8 cells formed an epithelial monolayer that maintained uninterrupted connections with the A7 epidermis and A9 genitalia (Supplementary Fig. 3).

**Figure 2:**
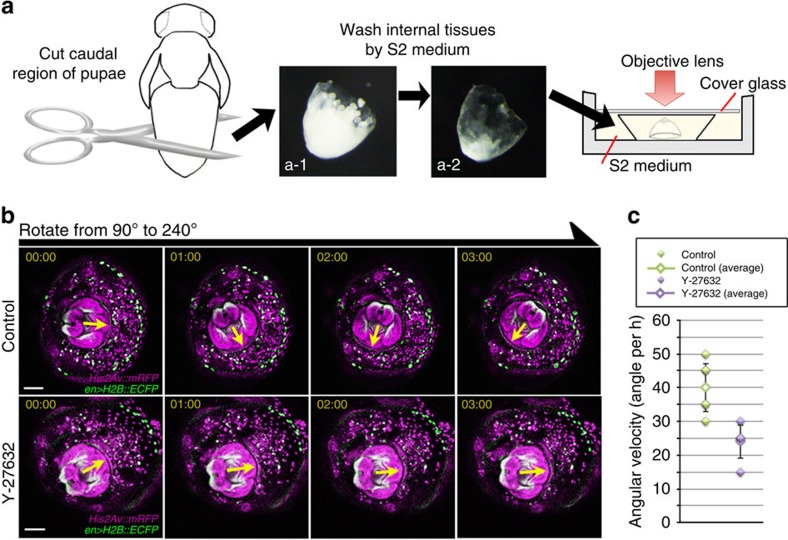
The autonomous epithelial movement is involved in genitalia rotation. (**a**) Schematic of the *ex vivo* culture experiment. The excised caudal region of pupae (**a**-1) was cleaned of internal tissues by pipetting with S2 medium (**a**-2), then placed into a culture tray with S2 medium. Time-lapse images were captured by a microscope. (**b**) Genitalia rotation occurred in the *ex vivo*-cultured genital disc (upper). The rotation was impaired by Y-27632 treatment (lower). Green: nuclei in the posterior of each segment, visualized by ECFP in the nucleus driven by *en-GAL4*. Magenta: *His2Av::mRFP*. Scale bar, 100 μm. (**c**) Graph showing the angular velocity of the *ex vivo*-cultured genital disc rotation, calculated for 2 h after the beginning of *ex vivo* culture. Error bars indicate s.d. Fly genotypes: *en-GAL4 UAS-H2B::ECFP/+; His2Av::mRFP/+* (**a**,**b**).

In some cases of epithelial deformation driven by cell intercalation, the movement of epithelial cells is autonomously induced without external support from other tissues, as long as each cell has the appropriate polarity. To investigate whether the A8a movement was controlled by an external force, as seen in the hindgut's attachment to the A10 analia^26^, or is autonomously controlled, we conducted an *ex vivo* genital disc rotation experiment. We dissected the caudal part of the pupal abdomen, leaving only the segments after A7, without detectable hindgut tissue (Fig. 2a). We then cultured these tissues and performed live imaging of the genital disc *ex vivo*. The genitalia in these samples continued to rotate (Fig. 2b). These findings suggested that the A8 tergite moves autonomously. We then examined whether Myo-II is involved in the autonomous A8 movement by performing the *ex vivo* experiment using Y-27632. Y-27632 inhibits the phosphorylation of myosin regulatory light chain by inhibiting the ROCK/Rho kinase activity. We observed that Y-27632 impaired the genitalia rotation in the *ex vivo* condition (Fig. 2b,c). Consistent with the rotational defect in the *sqh* dsRNA-expressing flies, the autonomous movement of A8a required Myo-II activity.

### LR asymmetric cell intercalation in epithelial movement

**Figure 3:**
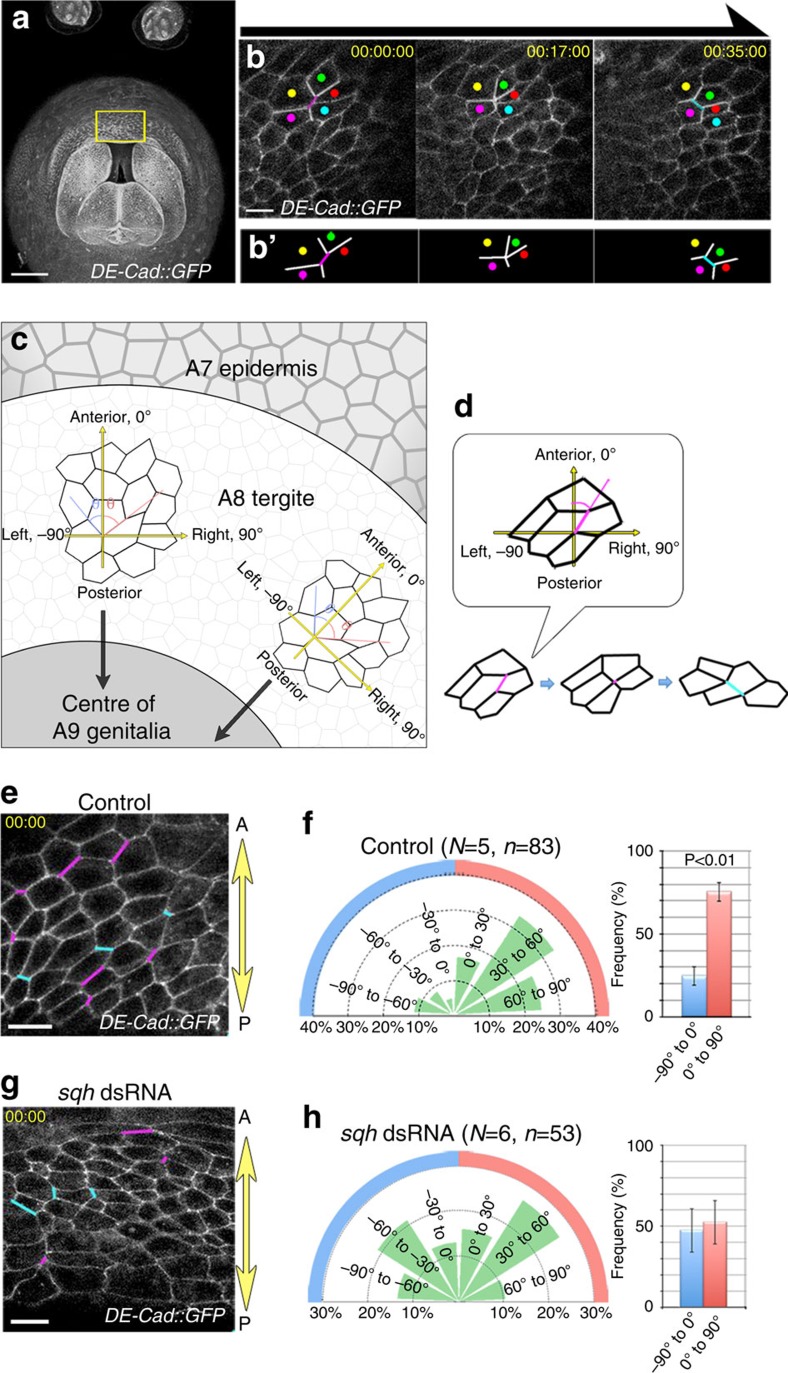
Myo-II-related LR asymmetric cell intercalation in A8a epithelial cells. (**a**) *DE-Cad::GFP* was observed at all the cell boundaries at 21 h APF. Yellow-boxed region was magnified in **b**. Scale bar, 50 μm. (**b**) Time-lapse images of epithelial cell rearrangement and migration in the A8a, visualized with *DE-Cad::GFP*. Schematic drawing represents the cell intercalation observed in the upper panels (**b**'). Scale bar, 10 μm. (**c**) Schematic drawing of the procedure for analysing the angle (*θ*) between the line formed by each cell boundary and the AP axis. The AP axis of A8a radiates in all directions from the centre of A9 to the A7 epidermis. Because the A8 tergite forms a ring-like structure surrounding the A9 genitalia at the pupal stage, the AP axis of A8 radiates in all directions from the centre of A9 to the A7 epidermis. The cell boundary angles were classified as being in the −90° to 0° (blue arc) or the 0° to 90° (red arc) range. (**d**) The angle (*θ*) formed between each cell boundary undergoing cell intercalation and the AP axis before remodelling was measured. (**e**,**g**) Cell boundaries visualized with *DE-Cad::GFP* in control (**e**) and *sqh* knockdown flies (**g**). The AP axis of the A8 (yellow arrow) was defined as the line from the A7 epidermis to the A9 genitalia. The angle (*θ*) formed between each cell boundary undergoing junction remodelling, followed by cell intercalation and the AP axis was measured. The cell boundary angles were classified as ranging from −90° to 0° (cyan) or 0° to 90° (magenta). Scale bar, 10 μm. (**f**,**h**) Rose diagrams depicting the percentage of cell intercalation axes in the A8a of control flies (**f**) and *sqh* dsRNA-expressing flies (**h**) at 30° intervals of the angle *θ* during cell movement. The percentage of cell boundaries (showing junction remodelling) with angle *θ* in the ranges indicated are determined. The frequency of cell intercalation was 13.7% (83/607 in five samples) in the control, and 7.3% (53/726 in six samples) in the *sqh* dsRNA-expressing flies. Right histograms show the percentage of cell intercalation axes in the ranges indicated. Bars indicate s.d. The numbers of flies and cell boundaries analysed are indicated as *N* and *n*, respectively. Fly genotype: *DE-Cad::GFP* (**a**,**b**,**e**,**f**), *DE-Cad::GFP; AbdB-GAL4 UAS-RedStinger/UAS-sqh dsRNA* (**g**,**h**).

Given the requirement for Myo-II activity in A8a movement, we expected that the epithelial cells in A8a would undergo cellular movement accompanied by convergent extension. We therefore observed the cellular dynamics during A8a movement using *DE-Cad::GFP* knock-in flies^27^ (Fig. 3a). We found that the A8a cells throughout the epithelium maintained adherens junctions with their neighbours during genitalia rotation, and underwent frequent junction remodelling, followed by cell intercalation, which allowed the cells to rearrange their positions (Fig. 3b,b' and Supplementary Movie 2). To identify the polarity of the cell intercalation, we examined the cell boundaries during the A8a cell directional movement, and measured the angle *θ* formed by the remodelled junctions with the AP axis (Fig. 3c,d). As shown in rose diagrams depicting the frequency of cell boundaries undergoing cell intercalation over 30° intervals, the remodelled cell boundaries tended to form a right oblique angle (*θ*, 0° to 90°) to the AP axis (Fig. 3e,f and Supplementary Movie 3). To determine whether this polarized cell intercalation was related to the Myo-II activity, we examined the polarity of the cell intercalation in flies expressing *sqh* dsRNA. Interestingly, *sqh* knockdown caused both a simultaneous decrease in the frequency of cell intercalation and a loss of the polarized orientation of cell intercalation (Fig. 3g,h and Supplementary Movie 4). These findings suggested that Myo-II contributes not only to the junctional contractility but also to the (right-biased) polarized remodelling in the A8a epithelium.

### Myo-II-related cell intercalation is directed by myoID

**Figure 4:**
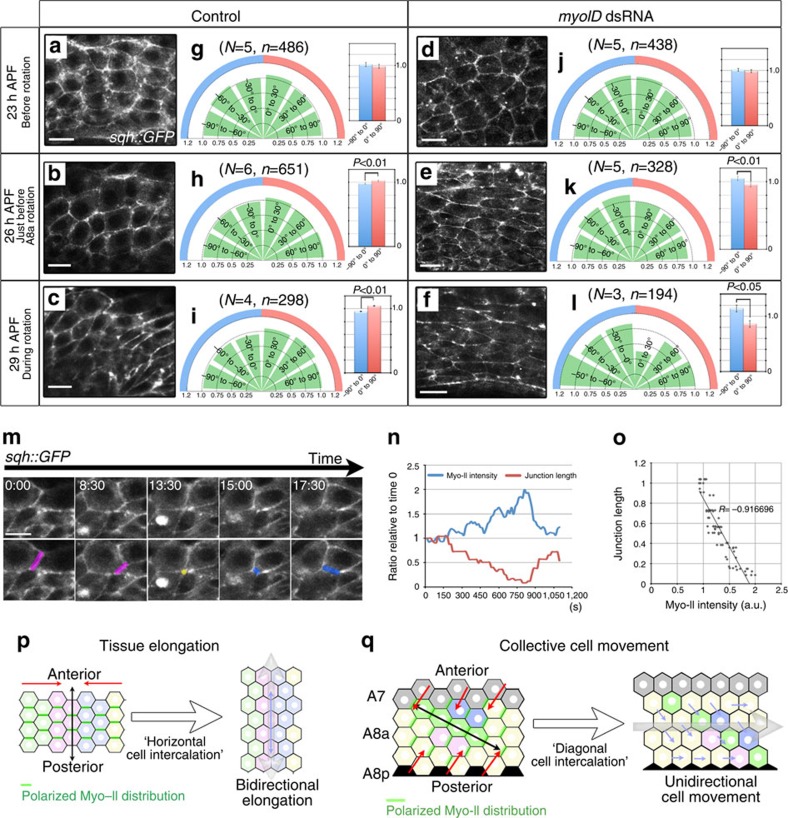
LR asymmetric Myo-II accumulation in A8a epithelial cells. (**a**–**f**) Myo-II protein observed by the GFP fluorescence of *sqh::GFP* in control (**a**–**c**) and *myoID* dsRNA-expressing flies (**d**–**f**), at 23 h APF (**a**,**d**), 26 h APF (**b**,**e**) and 29 h APF (**c**,**f**). Scale bar, 10 μm. (**g**–**l**) Rose diagrams showing the mean of the normalized *sqh::GFP* fluorescence intensities at cell boundaries in the A8a of control (**g**–**i**) and *myoID* knockdown flies (**j**–**l**), at 30° intervals of angle *θ*, at 23 h APF (**g**,**j**), 26 h APF (**h**,**k**) and 29 h APF (**i**,**l**). Right, histograms showing the mean of normalized intensities representing the indicated ranges of angle *θ*, and error bars indicating s.d. Number of flies, *N*; number of cell boundaries, *n*. (**m**–**o**) Cell intercalation tracked by *sqh:GFP* expression. The junction length (red) and Myo-II intensity (blue) are shown in **n**, and the inverse correlation between junction length and Myo-II intensity is plotted in **o**. Scale bar, 10 μm. Fly genotypes: *sqh*^*AX3*^*/Y; sqh::GFP* (a-c, g-i, and m-o), *sqh*^*AX3*^*/Y; sqh::GFP; AbdB-GAL4/UAS-myoID dsRNA* (**d**–**f** and **j**–**l**). (**p**,**q**) Schematic of germ-band elongation (**p**) and our model of collective cell migration (**q**), which are both mediated by cell intercalation. Red arrow: direction of contraction; black arrow: direction of elongation; green line: Myo-II distribution; blue arrow: direction of cellular movement; grey arrow: direction of global cellular flow.

Cell intercalation in various systems is induced by a planar polarized junctional contractility elicited by Myo-II, followed by junctional shortening^5^,^7^,^23^,^24^. Thus, we examined Myo-II's distribution in A8a epithelial cells. We determined the relative amount of Myo-II at each cell boundary using a *sqh::GFP* reporter construct^28^ (Fig. 4a–c). We previously reported that A8p starts rotating at 24 h APF, and A8a starts at 26–27 h APF^19^ (Fig. 6e). Thus, we determined the Myo-II distribution at 23 h (before rotation), 26 h (just before A8a rotation) and 29 h (during rotation) APF. Higher Myo-II levels were observed at cell boundaries forming a right oblique angle with the AP axis than at those forming a left oblique angle at 26 h (Fig. 4h) and 29 h APF (Fig. 4i), but not at 23 h APF (Fig. 4g). The observation of the LR asymmetrically polarized accumulation of Myo-II at cell boundaries just before rotation suggested that this polarization (right-biased) contributes to the induction of LR asymmetric intercalation (right-biased). To the best of our knowledge, this is the first time the LR asymmetric distribution of Myo-II at cell boundaries has been reported. To check the relationship between Myo-II distribution and cell intercalation, we determined the junction length and Myo-II intensity for junctions undergoing cell intercalation and for junctions that did not intercalate. In both cases, the junction length and Myo-II intensity were inversely correlated, although the actual values fluctuated (Fig. 4m–o, Supplementary Movie 5 and Supplementary Fig. 4). These data suggest that Myo-II accumulation causes junction shrinkage in A8a cells, whether or not the junction might undergo cell intercalation.

It is known that *myoID* contributes to the clockwise direction of genitalia rotation^21^. To examine whether the LR asymmetrically polarized Myo-II accumulation was involved in the directional movement of epithelial cells in A8a, we analysed the Myo-II distribution in flies expressing *myoID* dsRNA (Fig. 4d–f). In these flies, A8a moved counter-clockwise, whereas A8p moved in the clockwise (normal) direction, resulting in an apparent lack of genitalia rotation (Supplementary Fig. 5). The *myoID* reduction reversed the polarized distribution of Myo-II (to become left-biased) at 26 h (Fig. 4k) and 29 h APF (Fig. 4l), but not at 23 h APF (Fig. 4j), suggesting that *myoID* plays a role in determining the directional properties of the Myo-II polarization and cell intercalation. These data also indicated that the polarized accumulation of Myo-II in A8a does not depend on the prior movement of A8p.

These findings imply that LR asymmetrically polarized cell intercalation is involved in generating the directional force required for epithelial cellular movement. This role is distinct from the previously described function of cell intercalation in germ-band elongation, in which the accumulation of polarized Myo-II at the cell boundaries perpendicular to the elongation axis induces ‘horizontal cell intercalation' to elicit the bidirectional elongation of epithelial tissue along the AP axis (Fig. 4p)^5^,^23^. In genitalia rotation, the LR asymmetrically polarized Myo-II that is concentrated at the right oblique cell boundaries, with respect to the AP axis, induces ‘diagonal cell intercalation', resulting in oblique elongation with respect to the AP axis. The AP axis space interval in A8a is limited (A8a is sandwiched between A7 and A8p), and the A7 immobile layer flanking the anterior end of A8a provides spatial inhomogeneity with respect to the AP axis, allowing cells in A8a to undergo unidirectional movement perpendicular to the AP axis (Fig. 4q).

### Numerical simulation of collective cell movement

Our observations indicated that Myo-II-dependent LR asymmetrically biased cell intercalation was required to drive the directional movement of A8a cells. To evaluate this possibility, we used a mathematical vertex model^29^,^30^,^31^,^32^. This model is widely accepted as an accurate representation of epithelial cell sheet dynamics, incorporating the mechanical forces within cells and the cell intercalation between cells (see Supplementary Notes for the details of this model). The basic assumptions of the vertex model are as follows. Epithelial cells and their motions are represented by polygons and displacements of the vertices, respectively. Cell intercalation is represented as junctional remodelling that is implemented as reconnections of the line segments. The dynamics of the vertex positions are given by the balance between mechanical forces and frictional resistance representing the attachments with adjacent cells. The mechanical forces have the following four contributions: the hydrostatic pressure acting on cytoplasm in the cells, the tendency of the cell perimeter to be conserved, the boundary conditions of the cell sheet and the contraction force acting on each cell boundary. Although the first three contributions are defined essentially the same as in previous studies^30^,^33^, the last one includes novel aspects, as explained below. (For a more detailed explanation of our model, see the Supplementary Notes, in which all of the parameters we used in these simulations are indicated.)

**Figure 5:**
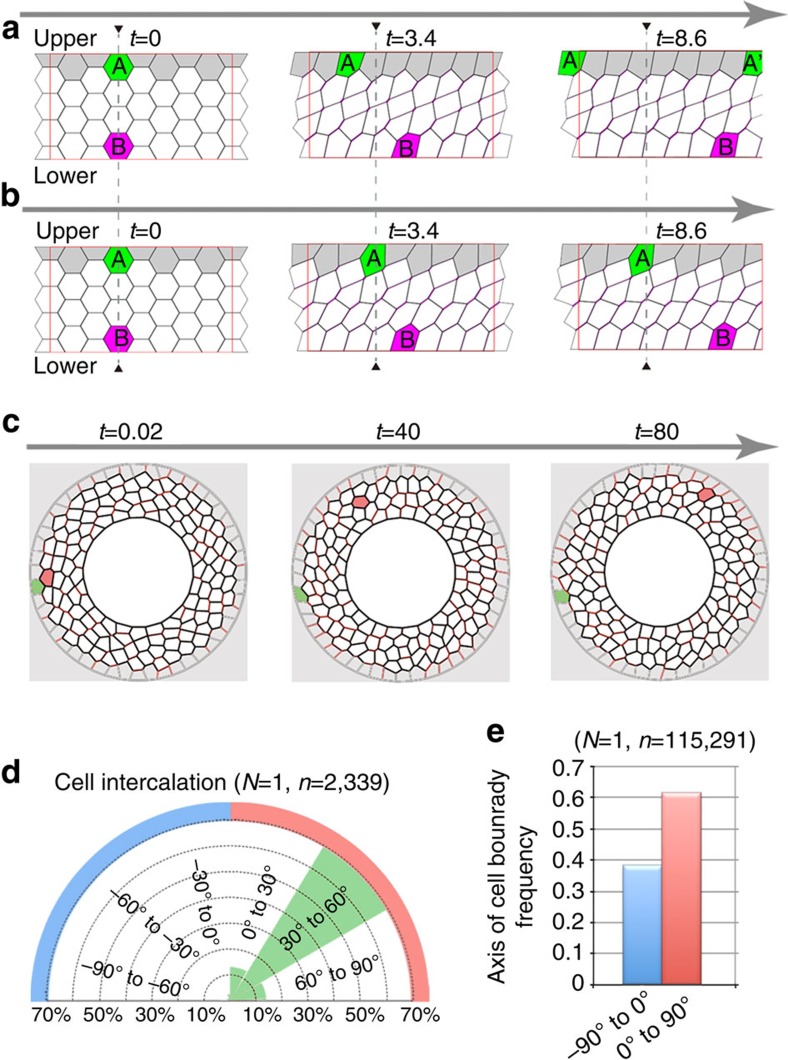
Numerical simulation of collective cell movement. (**a**,**b**) A directional dependence of the contraction forces on cell boundaries is assumed, in which cell boundaries inclining to the right have the largest contraction forces. The thickness of the magenta segments indicates the strength of the forces. As a guide, two cells are coloured magenta and green, and their original positions are indicated by black triangles and broken lines. The cells at the upper layer of the virtual epidermis are coloured grey. Numerical simulation of the condition in which the frictional coefficients at the upper and lower surfaces, denoted by *μ*_upper_ and *μ*_lower_, respectively, are equal (*μ*_upper_=*μ*_lower_=1.0) (**a**). Simulation of the condition in which *μ*_upper_=100.0 and *μ*_lower_=1.0 (**b**). The cells are mobile in both cases, although the whole sheet moves in one direction only in **b**. (**c**) Numerical simulation, from the present model reproducing the *in vivo* situation. We applied much greater friction on the outer (A7) border, *μ*_A7_=100.0, than on the other vertices, *μ*_i_=1.0. Cell boundaries are represented by lines ranging from black (lower tension) to red (higher tension). As a guide, two cells are coloured red and green. The outermost borders of the A8a and A8p are represented by the inner circle, and the A7 epidermis is indicated by the grey region. (**d**) Rose diagrams representing the percentage of cell intercalation axes at 30° intervals of angle *θ* in the virtual cells *in silico* (illustrated in **c**). (**e**) PCC was observed *in silico*. Frequency of cell boundaries with angle *θ* in the range −90° to 0° or 0° to 90° with respect to the AP axis *in silico* (demonstrated in **c**). Number of trials, *N*; number of cell boundaries, *n* (**d**,**e**).

To simulate the LR asymmetric polarity of Myo-II accumulation, we introduced a directional dependency to the strength *γ*_*ij*_ of the boundary contraction, such that the contractile force was strengthened specifically on the cell boundaries that inclined clockwise from the AP axis. Specifically, we set *γ*_*ij*_ as *γ*_*ij*_=0.5 (1+cos 2(*θ*_*ij*_–45°)) in which angle *θ*_*ij*_ was between the cell boundary *ij* and the AP axis (see Supplementary Notes for details). This setup for *γ*_*ij*_ implies that the contraction force is maximal when the segment is tilted +45° around the AP axis, whereas no contraction is imposed when it is tilted −45° around the AP axis. Numerical simulations showed that this enhancement of contraction on the inclined boundaries enables the cells to execute repeated junction remodelling and perpetual shear motion, with appropriate parameter values (Fig. 5a, Supplementary Fig. 8 and Supplementary Movie 6). Furthermore, if the frictional coefficient of the cell edges attached to the upper plate is very high compared with that of the lower plate, which represents the situation where the length of cell interfaces hardly changes in the upper region of the upper cells, the upper cells will not move, and only the lower cells will move to the right (Fig. 5b and Supplementary Movie 7). In mathematical terms, if each element in the system has some LR asymmetry and there is spatial inhomogeneity in some direction, the entire system can move in the direction perpendicular to that of the inhomogeneity^34^.

We next tested whether our numerical simulation accurately reflected the *in vivo* situation. To do this, we first rebuilt the model to include the circular cell boundary condition (Fig. 1b) and fluctuating junctional shrinkage, which are conditions found *in vivo* (Fig. 4n and Supplementary Fig. 4). The former was implemented by using two circles of different radii, which confine the virtual A8 epithelial cells (Fig. 5c). The outer circle represented the outer edge of the innermost layer of A7 cells, that is, the cells coloured grey in Fig. 5c and Supplementary Movie 8 are A7 cells. The inner circle represented the boundary between A8a and A8p. The fluctuating junctional shrinkage was incorporated by adding noise to the contraction forces acting on the cell boundaries (see Supplementary Notes for details). The defined AP axis was oriented radially from the centre of the inner circle, in a manner corresponding to the *in vivo* situation (Fig. 3c). We applied a high level of friction (*μ*_A7_) at the outer border to mimic the effect of the immobile A7 epidermis on the cells at the periphery of the virtual epidermis. Using this model, cells confined by the circular borders moved continuously to the right (with respect to the AP axis; Fig. 5c and Supplementary Movie 8), as observed *in vivo*. In addition, the numerical simulation showed that cell intercalation tended to occur at the right oblique cell boundaries (Fig. 5d,e), recapitulating the *in vivo* situation. Even if the angles specifying the direction of cell polarity were more broadly distributed, the qualitative results of the movement were the same as in the case where the direction of cell polarity was 45°, in the sense that the whole system moved to the right. In this case, the distribution of cell intercalation angles became broad, that is, left-biased cell intercalation also occurred, although with a lower frequency than right-biased cell intercalation.

### PCC plays a role in directional organ rotation

Our *in vivo* and *in silico* results indicated that the LR asymmetrical cell intercalation drives the directional movement of the epithelial cells in A8a. However, it was still unclear how the cells in A8a maintain their polarity. To address this question, we referred to the decision principle for the direction of the twist of the *Drosophila* embryonic hindgut^35^. Hindgut epithelial cells adopt a LR asymmetric cell shape within their plane, termed PCC, and previous numerical simulation analysis revealed that this PCC is sufficient to induce the directional twisting force for hindgut asymmetry^35^. To assess whether PCC is involved in genitalia rotation, we first examined whether PCC was represented in our numerical simulation. According to the previous report on *Drosophila* hindgut, not only the cell shape but also the *D*E-Cad distribution and centrosome position show chirality in epithelial cells^35^. In this study, however, we only analysed the eventual phenotype, the PCC, by measuring the angle (*θ*) between the virtual epithelial cell boundaries and the AP axis, as described previously^35^. The results revealed that cell boundaries forming a right oblique angle *θ* (0° to 90°) with the AP axis became more frequent than those forming a left oblique angle (−90° to 0°) as the simulation progressed (Fig. 5e). Therefore, we speculated that the A8a epithelial cells also have PCC, which helps to determine the tissue's rotational direction.

**Figure 6:**
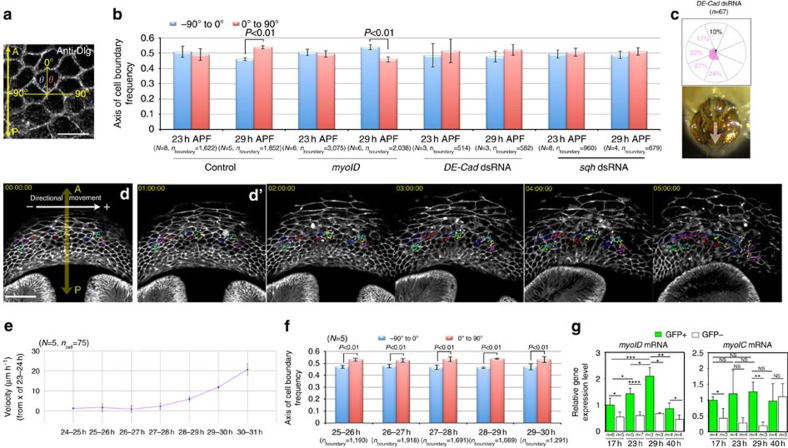
PCC plays a role in the rotational direction of genitalia. (**a**) The angle between the cell boundary (dashed line), labelled for Discs Large (Dlg; white) and the AP axis (yellow arrow) was defined as *θ* (blue and red arcs). Scale bar, 10 μm. (**b**) Frequency of cell boundaries with an angle *θ* of −90° to 0° or of 0° to 90° to the AP axis in controls, *myoID*, *DE-Cad* knockdown and *sqh* knockdown flies at 23 and 29 h APF. (**c**) Rose diagrams indicating the frequency of external genitalia orientation in adult male *DE-Cad* knockdown flies. White arrow indicates the direction from the analia to the external genitalia. (**d**) First image in a time-lapse series showing genitalia rotation. Cells were assigned different numbers and colours, and their rightward velocity with respect to the AP axis was tracked (yellow arrow). Rightward movement was defined as positive unidirectional motion (white arrow). A time-lapse series for **d** is shown in **d**'. Scale bar, 50 μm. (**e**) Velocity of rightward cellular movement with respect to the AP axis. (**f**) Frequency of cell boundaries forming an angle *θ* with the AP axis at each time point, in the control flies analysed in **d**. (**g**) Gene expression levels for *myoID* and *myoIC* were analysed by qRT–PCR. Green bars are GFP-positive cells and white bars are GFP-negative cells in the dissected tissue. *P<0.05, **P<0.01, ***P<0.001, ****P<0.0001. Error bars indicate s.d. (**b**,**e**,**f**,**g**). Number of flies, *N*; number of cell boundaries, *n*_boundary_ (**b**,**e** and **f**); number of experimental replicates, *n* (**g**). Fly genotypes: *UAS-Dcr2/+; AbdB-GAL4 UAS-RedStinger/+* (**a**,**b**, Control), *myo31DF*^*k2*^ (**b**, *myoID*), *UAS-Dcr2/UAS-shg dsRNA; AbdB-GAL4 UAS-RedStinger/+* (**b**,**c**, *DE-Cad* dsRNA), *UAS-Dcr2/UAS-sqh dsRNA; AbdB-GAL4 UAS-RedStinger/+* (**b**, *sqh* dsRNA) *Nrg::GFP*^*G305*^*/Y* (**d**–**f**). NS, not significant.

Consistent with the simulation, we observed a right-biased PCC during genitalia rotation (29 h APF) in control flies (Fig. 6a,b, control). To confirm this scenario from another point of view, we measured the distribution of cell-boundary angles in a homozygous *myoID* mutant, which exhibits heterotaxy in its embryonic hindgut and in the looping of its adult spermiduct^21^,^36^. We found that the distribution of boundary angles in the A8a epithelial cells of the mutant was reversed from that of control flies (Fig. 6b, *myoID*), suggesting that PCC is indeed a property of the A8a epithelial cells and that it is related to the moving direction of these cells. The only known genetic manipulation reported to abolish PCC is the reduction of *D*E-Cad in embryonic hindgut twisting^35^. We found that the LR asymmetry in A8a was lost in *D*E-Cad knockdown flies (Fig. 6b, *DE-Cad* dsRNA), in which the genitalia rotation was also impaired (Fig. 6c).

Based on the PCC-forming process in our numerical simulation, the establishment of PCC may depend on the LR asymmetric contraction and cell intercalation. To examine this possibility, we analysed the PCC in *sqh* knockdown flies, in which polarized cell intercalation was impaired. PCC was abolished in the *sqh* dsRNA-expressing flies (Fig. 6b, *sqh* dsRNA), suggesting that PCC may be a consequence of the LR asymmetric contraction and intercalation. This mimicking of another *in vivo* observation by the numerical simulation helped validate our model, in which the LR asymmetry of cells is related to the direction of cellular movement.

To check whether the LR asymmetry in the A8a cell plane was a cause or a consequence of the directional movement of the cells, we determined the timing with which the PCC reflecting the LR-biased contraction was formed, namely, whether it was just before or after the onset of epithelial movement. We compared the changes over time in the PCC and in the directional movement of A8a cells using time-lapse imaging (Fig. 6d and Supplementary Movie 9). The results revealed that PCC was present just before the A8a cells began to move, and persisted during A8a cell movement (Fig. 6e,f).

Finally, to check whether the timing of PCC formation is related to *myoID* expression, we investigated the *myoID* mRNA expression levels in A8a cells before and during rotation. Using FACS (fluorescence activated cell sorting), we sorted the green fluorescent protein (GFP)-positive cells from flies expressing GFP driven in A8a cells by the *AbdB-GAL4*^*LDN*^ driver at two time points before rotation (17 and 23 h APF) and one time point during rotation (29 h APF) and found that the level of *myoID* mRNA increased over time (Fig. 6g). We also analysed the level of *myosin 61F* (*myoIC)* mRNA, which is hypothesized to act as a functional antagonist of myoID^37^, but found no difference among these stages (Fig. 6g). Furthermore, when we examined the levels of *myoID* and *myoIC* mRNAs at a later time point, after the rotation (40 h APF), only *myoID* was significantly reduced compared with its levels during rotation (29 h APF; Fig. 6g). Taken together, the *myoID* level increases to exceed a threshold to overcome the antagonistic effect of *myoIC* or other unidentified antagonists, which may be involved in the formation of PCC, and the conclusion of movement may also depend on the *myoID* mRNA level.

In hindgut twisting, PCC is observed before the beginning of cell deformation, and then the twisting occurs as a consequence of the cancellation of PCC. Once cells assume a symmetrical hexagonal shape in their plane at the end of hindgut twisting, no more twisting is observed^35^. In contrast, in genitalia rotation, the LR asymmetry in the cell plane was continuously observed as the rotation progressed. We speculate that the persistent LR asymmetric polarized contraction/cell intercalation and the subsequent PCC linked with *myoID* expression both play roles in the continuous and directional cellular movement in the A8a epithelial tissue. Using our *in vivo* observations and *in silico* model (a ring shape with no leading edge), we revealed a mechanism for collective cell movement occurring during epithelial morphogenesis that is different from the conventional guidance-mediated directional cell movement.

## Discussion

Based on our theoretical and *in vivo* data, we here propose a model for collective cell movement in which integration of the individual LR asymmetry of epithelial cells and polarized cell intercalation is sufficient to organize the global cellular flow of epithelial tissue. Our observations indicated that in the process of clockwise A8a epithelial movement, the strongest polarized accumulation of Myo-II is followed by the polarized intercalation, and then by PCC in A8a. Although myoID appears to act upstream of Myo-II, given that *myoID* knockdown reversed the Myo-II distribution, Myo-II was still polarized even in the opposite boundaries in this fly, suggesting the existence of unidentified machinery controlling the polarized accumulation of Myo-II just before A8a movement. Germ-band elongation in *Drosophila* embryogenesis requires the concerted shrinkage of all the cell boundaries perpendicular to the AP axis, as well as the polarized accumulation of Myo-II (refs 5, 23, 31). In that system, it was shown that the planar polarized contractile flow of Myo-II depends on polarized fluctuations in E-Cad at the periphery of the medial apical actomyosin network; these E-Cad fluctuations along the AP axis attract the medial actomyosin flow, which then induces a local deposition of Myo-II at the junction plate^38^. Thus, further studies investigating the movement of actomyosin in A8a just before *Drosophila* genitalia rotation will be necessary to elucidate how Myo-II's distribution is polarized in this system.

Another area for future study is the identification of the cue(s) that induce(s) directional cellular movement, which may involve the Myo-II-dependent establishment of cell-shape chirality. As we mentioned above, myoID is an upstream factor determining the appropriate polarization of Myo-II. Our detection of Myo-II polarity linked with the increased expression of *myoID* suggests that the establishment of LR asymmetry is related to the initiation cues for directional cellular movement. An upstream candidate of *myoID* increase is *AbdB*, which is reported to serve as a transcriptional activator of *myoID* during genitalia rotation^22^; however, the changes in *AbdB* level over time have not been reported. A recent study demonstrated that Toll-2, Toll-6 and Toll-8 act in combination to direct planar polarity and polarized cell rearrangements during convergent extension^39^. Thus, it would be intriguing to investigate the contribution of Toll family receptors to PCC (and LR asymmetric intercalation) in genitalia rotation.

We previously reported that apoptosis contributes to the movement of A8a during the morphogenesis of male terminalia^19^, but how apoptosis induces A8a movement remained unclear. Apoptotic cells are extruded from epithelial sheets via apical constriction, in which actin–myosin is reported to form contractile bundles in a two-dimensional network underling the apical cortex^40^. This apical constriction is thought to be the source of the apoptotic force^41^. Myo-II dynamics are reported to be affected by changes in mechanical tension. An experiment in which mechanical stress was genetically manipulated by ectopic perturbations of cell numbers in the wing disc showed that the Myo-II distribution in the surrounding cells was reorganized non-autonomously, suggesting that local mechanical stress (by apoptotic force in our case) can affect the Myo-II polarization in surrounding cells^42^. Another recent study indicated that apoptotic cells induce a non-autonomous increase in tissue tension by stabilizing the apical cortical Myo-II in the surrounding tissue, resulting in leg epithelium folding^43^. Further experiments are needed to address whether or how apoptosis contributes to reorganizing the global pattern of Myo-II distribution during the asymmetrical deformation of epithelial cell sheets in *Drosophila* genitalia rotation.

The investigation of collective cell movement during various morphogenetic processes has revealed a variety of cellular behaviours that contribute to the shaping of tissues and organs during homeostasis, wound repair and disease. Much of the research on collective cell movement has focused on characterizing the cells in the leading edge. For example, in the zebrafish lateral line organ, only a small number of chemokine-responsive cells at the leading edge are required to direct the polarity of the cells in the entire tissue^44^. In *Drosophila* oogenesis, border cells migrate as a cluster during late oogenesis and form a leading edge in response to chemotactic cues^45^,^46^. Another example is epithelial sheet movement during wound repair or closure, which uses a contractile actomyosin ring at the leading edge^47^. In contrast, mechanisms by which epithelial cells that fill a confined space, or by which a cluster of epithelial cells without a leading edge, move synchronously in a single direction to induce large tissue distortion with no net translocation, have remained unclear. The collective movement of epithelial cells during genitalia rotation is a suitable model system for uncovering these mechanisms, because the cells in A8a maintain a ring shape during this process. Our results in this study reveal one possible mechanism for this type of collective cell movement. Egg chamber rotation in *Drosophila* oogenesis is another model system^14^,^15^,^16^,^17^. However, while the direction of genitalia rotation is genetically determined, individual egg chambers rotate in either a clockwise or counter-clockwise direction, raising the possibility that the underlying mechanism involves a stochastic process. On the other hand, the follicle's closed topology results in the collective migration of epithelial cells that fill a confined space. Therefore, a comparative study of these systems may uncover common mechanisms for the synchronous motion of epithelial cells in a confined space that lack a leading edge.

Finally, the classical paradigm of cell migration suggests that the dynamic remodelling of integrin-based cell-substrate adhesion provides both movement and directionality, and it was recently reported that integrin superfamily mutants exhibit defective male genitalia rotation^48^. These findings suggest that future studies should also investigate interactions between the cell intercalation during genitalia rotation and the basal adhesion system. Our model, which proposes that the dynamics within apical cell planes are sufficient for generating the driving force required for cellular movement during epithelial tissue morphogenesis, may provide mechanistic insight into other developmental processes involving epithelial morphogenesis.

## Methods

### *Drosophila* strains

All fly stocks were maintained on standard fly medium at 25 °C. Fly strains and their sources were as follows: *en-GAL4, UAS-shg dsRNA* (BL32904)*, UAS-sqh dsRNA* (BL31542), *UAS-Dcr2 (II)*, *UAS-GFP (II)*, *Histone2Av::mRFP (His2Av::mRFP) (II)*, *Histone2Av::mRFP (His2Av::mRFP) (III)*, *UAS-RedStinger (III)* (Bloomington *Drosophila* Stock Center); *emc::GFP*^*YB0040*^ (ref. 49); *Nrg::GFP*^*G305*^ (ref. 50); *sqh*^*AX3*^*/Y; sqh::GFP/CyO*^28^; *DE-Cad::GFP*^27^; *AbdB-GAL4*^*LDN*^ (ref. 51); *myo31DF*^*k2*^ (ref. 21), *UAS-myoID dsRNA*^21^; *UAS-Histone2B-ECFP (UAS-H2B::ECFP)*^52^.

### *Ex vivo* culture experiment

The *ex vivo* culture experiment was performed as illustrated in Fig. 2a. The caudal part of the pupal abdomen that included segments following the A7 area was dissected, washed with S2 medium by pipette to remove fat bodies and placed into *Drosophila* S2 medium (Life Technologies). Time-lapse images were captured with a stereomicroscope (M205FA, Leica).

### Immunohistochemistry

Staged pupal genital discs were dissected in PBS and fixed for 20 min with 4% paraformaldehyde/PBS. The samples were washed six times for 5 min each with 0.1% (vol/vol) Triton X-100/PBS and incubated overnight at 4 °C with the primary antibody and 5% (vol/vol) donkey serum. The samples were then washed and incubated for 2 h at room temperature with the secondary antibody and 5% (vol/vol) donkey serum, mounted with 70% glycerol and observed by confocal microscopy (TCS SP5 and SP8; Leica). The primary antibodies were mouse anti-Dlg 1/500 (DLG1, DSHB), anti-engrailed 1/20 (4D9, DSHB), rat anti-*D*E-cad 1/50 (DCAD2, DSHB) and rabbit anti-GFP 1/500 (598, MBL). The secondary antibodies were donkey anti-mouse or anti-rabbit IgG conjugated to Alexa-488 or Alexa-555 (A21202, A21206, A31570, A31572, Molecular Probes). The nuclei were labelled with 20 μg ml^−1^ Hoechst 33342 (H1399, Molecular Probes).

### Film analysis

Time-lapse images were captured by confocal microscopy (TCS SP5 and SP8; Leica). The images were converted to a projection image using Leica LAS. Cell boundaries showing cell intercalation were manually tracked using ImageJ. Images were taken every 2 min, and cell boundaries showing cell intercalation were analysed within 50 min. The angle (*θ*) formed between each cell boundary undergoing cell intercalation and the AP axis before remodelling was measured. Because the A8 tergite forms a ring-like structure surrounding the A9 genitalia at the pupal stage, the AP axis of A8a radiates in all directions from the centre of A9 to the A7 epidermis (indicated in Fig. 3c). To determine the speed of the moving cells in A8, 15 individual cells were traced in each fly (*N*=5) using image analysis software (ImageJ) that included a tracking algorithm, to obtain the coordinates of cells on the ventral side of A8 every 2 min. The distance of movement between frames along a line perpendicular to the AP axis in a rightward direction was determined, and the mean velocity was calculated. The genotype of the flies used was *Nrg::GFP*^*G305*^.

### Calculation of the *sqh::GFP* intensity

Optical sections of the A8 epithelium were captured by confocal microscopy (TCS SP5 and SP8; Leica). The images were converted to projection images using Leica LAS. The mean signal intensity along each cell boundary was calculated using ImageJ (http://rsb.info.nih.gov/ij/). Cell boundary images from more than three regions of A8 in each individual were analysed. To normalize the antibody staining and s*qh::GFP* intensity among discs, the ratio of the intensity of each cell boundary to the mean of all the cell boundaries was calculated. The angle *θ* formed between each cell boundary and the AP axis was also measured from these images, and the correlation between the angle *θ* and the normalized intensity of s*qh::GFP* was calculated. The AP axis of A8a radiates in all directions from the centre of A9 to the A7 epidermis. The means of the normalized s*qh::GFP* intensities at cell boundaries sorted according to the indicated range of angle *θ* were calculated. The mean normalized intensity of *sqh::GFP* at the cell boundaries for each 30° interval was calculated and shown in rose diagrams. To compare the laterally biased distribution of *sqh::GFP* statistically, the means of its normalized intensity at cell boundaries with an angle *θ* of −90 to 0° and 0 to 90° were calculated.

### Analysis of LR asymmetry within the cell plane

The planar cell chirality of the cell shape in A8a was determined by the distribution frequency of cell boundaries with angle *θ* (indicated in Fig. 6a). Cell boundary images from more than three regions of A8a in each individual were analysed. The cell boundaries of the A8a epithelial cells were labelled with an anti-Dlg antibody or with *Nrg::GFP*. To identify the cell domains and their boundaries in an epithelial sheet, we performed image segmentation using the watershed transform, with ‘Watershed Components', a built-in function of Mathematica version 8.0. This function gives lists of pixels that specify the cell domains (catchment basins) and cell boundaries (watershed lines). The markers (seeds) for the watershed segmentation are entered by hand. The vertices of each cell are defined by the points where three or four watershed lines meet. The angle (angle *θ*) between the line formed by each cell boundary and the AP axis was determined with Mathematica. The AP axis of A8a radiates in all directions from the centre of A9 to the A7 epidermis. To analyse the PCC, the mean frequencies of cell boundaries with the angle *θ* of −90 to 0° and 0 to 90° were calculated. Standard errors among the average frequencies of these two bundles obtained from the indicated number of boundaries (*n*_boundary_) from the indicated number of flies (*N*) are shown by error bars.

### Statistical analysis

Significant differences were identified with an unpaired Student's *t*-test. Error bars in all graphs indicate s.d. (standard deviation). Significance was accepted at *P*<0.05. *P*-values are indicated as *P*.

### A8a cell preparation for FACS

For FACS analysis, 30–40 male pupae at each time point (17, 23, 29 and 40 h APF at 25 °C, *UAS-GFP/+; AbdB-GAL4/+*) were dissected and washed in PBS to obtain the caudal region containing segments A7–A10. The samples were transferred into 200 μl of 0.05% Trypsin-EDTA (Gibco), incubated for 30 min at room temperature, and then completely dissociated into single cells by pipetting. After centrifugation at 1,000 r.p.m. for 5 min (at 4 °C), the cells were washed in 1% BSA/PBS and resuspended in staining solution. The cells were stained with propidium iodide (Dojindo) at a final concentration of 1 μg ml^−1^ to eliminate dead cells, followed by incubation at 4 °C for 5 min. After three washes (with 1% BSA/PBS), the cell mixture was filtered through a 35-μm pore-size cell strainer (BD Falcon) to remove tissue debris. Using a Cell Sorter SH800 (Sony), the fractions of GFP-negative and GFP-positive cells were separated and collected.

### Quantitative reverse transcription–PCR (qRT–PCR) analysis

Total RNA from the cells collected by FACS was prepared using TRIzol reagent (Invitrogen) followed by reverse transcription using the PrimeScript RT reagent Kit (TaKaRa). qRT–PCR was performed on an ABI PRISM 7500 Real-Time PCR System (Applied Biosystems) using SYBR Premix Ex TaqII (TaKaRa). The level of the mRNA of interest was normalized to the *rp49* expression level. The fold change was the gene expression level relative to the level of the control cell population (17 h APF GFP-positive cells). The primers used were:

*myoID* forward primer:5′-GAAGCTGGAGTGCAGGACTT-3′

*myoID* reverse primer:5′-GATGGATCCGTTTTGGAATC-3′

*myoIC* forward primer:5′-CCAATCCCAAACTCCAAACTC-3′

*myoIC* reverse primer:5′-CTCGAGGAGCACAAAGTCCT-3′

*rp49* forward primer:5′-CGGATCGATATGCTAAGCTGT-3′

*rp49* reverse primer:5′-CGACGCACTCTGTTGTCG-3′.

## Additional information

**How to cite this article:** Sato, K. *et al.* Left–right asymmetric cell intercalation drives directional collective cell movement in epithelial morphogenesis. *Nat. Commun.* 6:10074 doi: 10.1038/ncomms10074 (2015).

## Supplementary Material

Supplementary Information Supplementary Figures 1-9, Supplementary Notes 1-4 and Supplementary References

Supplementary Movie 1 Male genitalia rotation in *His2Av::mRFP/+; AbdB-GAL4 UAS-H2B::ECFP/+*. Ventral is up, and images were acquired every 5 min. Movie was taken with a 20× objective lens.

Supplementary Movie 2 Rearrangement and migration of epithelial cells in *DE-Cad::GFP* flies. Anterior is up, and images were acquired every 15 sec. Movie was taken with a 20× objective lens.

Supplementary Movie 3 Junction shrinkage and remodeling at the cell boundaries with angle *θ* in the range -90° to 0° (cyan) or 0° to 90° (magenta) to the AP axis in the A8 tergite of control flies. Anterior is up, and images were acquired every 2 min. Movie was taken with a 20× objective lens. Fly genotype: *DE-Cad:GFP*.

Supplementary Movie 4 Junction shrinkage and remodeling at the cell boundaries with angle *θ* in the range -90° to 0° (cyan) or 0° to 90° (magenta) to the AP axis in the A8 tergite of a Myo-II RNAi fly. Anterior is up, and images were acquired every 2 min. Movie was taken with a 20× objective lens. Fly genotype: *DE-Cad::GFP; AbdB-GAL4 UAS-RedStinger/UAS-sqh dsRNA*.

Supplementary Movie 5 Junction shrinkage and remodeling at cell boundaries in the A8 tergite in a *sqh^AX3^/Y; sqh:GFP* fly. Anterior is up, and images were acquired every 15 sec. Movie was taken with a 20× objective lens.

Supplementary Movie 6 Numerical simulation of the vertex model in the flat boundary case. The friction coefficients of vertices are the same, i.e., *μ*_upper_ = *μ*_lower_ = *μ*_i_ = 1.0.

Supplementary Movie 7 Numerical simulation of the vertex model in the flat boundary case. The friction coefficients of the vertices on the border of the cell sheet are much larger than those of the others, i.e. *μ*_upper_ = 100.0, *μ*_lower_ = 1.0.

Supplementary Movie 8 Numerical simulation of the vertex model in the circular boundary case with fluctuation in line tension. The friction coefficients of the vertices on the border of the cell sheet are much larger than those of the others.

Supplementary Movie 9 Relationship between unidirectional movement and PCC over time. Cells were assigned different numbers and colors, and their rightward velocity was tracked with respect to the AP axis in a *Nrg::GFP^G305^/Y* fly. Ventral is up, and images were acquired every 5 min. Movie was taken with a 20× objective lens.

## References

[b1] Tada M. & Heisenberg C. P. Convergent extension: using collective cell migration and cell intercalation to shape embryos. Development 139, 3897–3904 (2012). 23048180 10.1242/dev.073007

[b2] Shih J. & Keller R. Cell motility driving mediolateral intercalation in explants of *Xenopus laevis*. Development 116, 901–914 (1992). 1295743 10.1242/dev.116.4.901

[b3] Sepich D. S. *et al.* Role of the zebrafish trilobite locus in gastrulation movements of convergence and extension. Genesis 27, 159–173 (2000). 10992326 10.1002/1526-968x(200008)27:4<159::aid-gene50>3.0.co;2-t

[b4] Lienkamp S. S. *et al.* Vertebrate kidney tubules elongate using a planar cell polarity-dependent, rosette-based mechanism of convergent extension. Nat. Genet. 44, 1382–1387 (2012). 23143599 10.1038/ng.2452 PMC4167614

[b5] Bertet C., Sulak L. & Lecuit T. Myosin-dependent junction remodelling controls planar cell intercalation and axis elongation. Nature 429, 667–671 (2004). 15190355 10.1038/nature02590

[b6] Nishimura T., Honda H. & Takeichi M. Planar cell polarity links axes of spatial dynamics in neural-tube closure. Cell 149, 1084–1097 (2012). 22632972 10.1016/j.cell.2012.04.021

[b7] Harris T. J., Sawyer J. K. & Peifer M. How the cytoskeleton helps build the embryonic body plan: models of morphogenesis from *Drosophila*. Curr. Topics Dev. Biol. 89, 55–85 (2009). 10.1016/S0070-2153(09)89003-0 19737642

[b8] Lecuit T. & Yap A. S. E-cadherin junctions as active mechanical integrators in tissue dynamics. Nat. Cell Biol. 17, 533–539 (2015). 25925582 10.1038/ncb3136

[b9] Tamada M., Farrell D. L. & Zallen J. A. Abl regulates planar polarized junctional dynamics through beta-catenin tyrosine phosphorylation. Dev. Cell 22, 309–319 (2012). 22340496 10.1016/j.devcel.2011.12.025 PMC3327890

[b10] Collinet C., Rauzi M., Lenne P. F. & Lecuit T. Local and tissue-scale forces drive oriented junction growth during tissue extension. Nat. Cell Biol. 17, 1247–1258 (2015). 26389664 10.1038/ncb3226

[b11] Friedl P. & Gilmour D. Collective cell migration in morphogenesis, regeneration and cancer. Nat. Rev. Mol. Cell Biol. 10, 445–457 (2009). 19546857 10.1038/nrm2720

[b12] Ridley A. J. *et al.* Cell migration: integrating signals from front to back. Science 302, 1704–1709 (2003). 14657486 10.1126/science.1092053

[b13] Heller E., Kumar K. V., Grill S. W. & Fuchs E. Forces generated by cell intercalation tow epidermal sheets in mammalian tissue morphogenesis. Dev. Cell 28, 617–632 (2014). 24697897 10.1016/j.devcel.2014.02.011 PMC4041280

[b14] Haigo S. L.. & Bilder D. Global tissue revolutions in a morphogenetic movement controlling elongation. Science 331, 1071–1074 (2011). 21212324 10.1126/science.1199424 PMC3153412

[b15] Bilder D. & Haigo S. L. Expanding the morphogenetic repertoire: perspectives from the *Drosophila* egg. Dev. Cell 22, 12–23 (2012). 22264728 10.1016/j.devcel.2011.12.003 PMC3266552

[b16] Viktorinova I. & Dahmann C. Microtubule polarity predicts direction of egg chamber rotation in *Drosophila*. Curr. Biol. 23, 1472–1477 (2013). 23831293 10.1016/j.cub.2013.06.014

[b17] Cetera M. *et al.* Epithelial rotation promotes the global alignment of contractile actin bundles during *Drosophila* egg chamber elongation. Nat. Commun. 5, 5511 (2014). 25413675 10.1038/ncomms6511 PMC4241503

[b18] Keisman E. L., Christiansen A. E. & Baker B. S. The sex determination gene doublesex regulates the A/P organizer to direct sex-specific patterns of growth in the *Drosophila* genital imaginal disc. Dev. Cell 1, 215–225 (2001). 11702781 10.1016/s1534-5807(01)00027-2

[b19] Kuranaga E. *et al.* Apoptosis controls the speed of looping morphogenesis in *Drosophila* male terminalia. Development 138, 1493–1499 (2011). 21389055 10.1242/dev.058958

[b20] Suzanne M. *et al.* Coupling of apoptosis and L/R patterning controls stepwise organ looping. Curr. Biol. 20, 1773–1778 (2010). 20832313 10.1016/j.cub.2010.08.056 PMC4516037

[b21] Speder P., Adam G. & Noselli S. Type ID unconventional myosin controls left-right asymmetry in *Drosophila*. Nature 440, 803–807 (2006). 16598259 10.1038/nature04623

[b22] Coutelis J. B. *et al.* *Drosophila* left/right asymmetry establishment is controlled by the Hox gene abdominal-B. Dev. Cell 24, 89–97 (2013). 23328400 10.1016/j.devcel.2012.11.013

[b23] Zallen J. A. & Wieschaus E. Patterned gene expression directs bipolar planar polarity in *Drosophila*. Dev. Cell 6, 343–355 (2004). 15030758 10.1016/s1534-5807(04)00060-7

[b24] Levayer R., Pelissier-Monier A. & Lecuit T. Spatial regulation of Dia and Myosin-II by RhoGEF2 controls initiation of E-cadherin endocytosis during epithelial morphogenesis. Nat. Cell Biol. 13, 529–540 (2011). 21516109 10.1038/ncb2224

[b25] Chen E. H. & Baker B. S. Compartmental organization of the *Drosophila* genital imaginal discs. Development 124, 205–218 (1997). 9006081 10.1242/dev.124.1.205

[b26] Coutelis J. B., Petzoldt A. G., Speder P., Suzanne M. & Noselli S. Left-right asymmetry in *Drosophila*. Semin. Cell Dev. Biol. 19, 252–262 (2008). 18328746 10.1016/j.semcdb.2008.01.006

[b27] Huang J., Zhou W., Dong W., Watson A. M. & Hong Y. From the cover: directed, efficient, and versatile modifications of the *Drosophila* genome by genomic engineering. Proc. Natl Acad. Sci. USA 106, 8284–8289 (2009). 19429710 10.1073/pnas.0900641106 PMC2688891

[b28] Royou A., Sullivan W. & Karess R. Cortical recruitment of nonmuscle myosin II in early syncytial *Drosophila* embryos: its role in nuclear axial expansion and its regulation by Cdc2 activity. J. Cell Biol. 158, 127–137 (2002). 12105185 10.1083/jcb.200203148 PMC2173028

[b29] Nagai T. & Honda H. A dynamic cell model for the formation of epithelial tissue. Philos. Mag. B 81, 699–719 (2001).

[b30] Farhadifar R., Roper J. C., Aigouy B., Eaton S. & Julicher F. The influence of cell mechanics, cell-cell interactions, and proliferation on epithelial packing. Curr. Biol. 17, 2095–2104 (2007). 18082406 10.1016/j.cub.2007.11.049

[b31] Rauzi M., Verant P., Lecuit T. & Lenne P. F. Nature and anisotropy of cortical forces orienting *Drosophila* tissue morphogenesis. Nat. Cell Biol. 10, 1401–1410 (2008). 18978783 10.1038/ncb1798

[b32] Nagai T. & Honda H. A dynamic cell model for the formation of epithelial tissues. Philos. Mag. B 81, 699–719 (2001).

[b33] Fletcher A. G., Osborne J. M., Maini P. K. & Gavaghan D. J. Implementing vertex dynamics models of cell populations in biology within a consistent computational framework. Prog. Biophys. Mol. Biol. 113, 299–326 (2013). 24120733 10.1016/j.pbiomolbio.2013.09.003

[b34] Sato K., Hiraiwa T. & Shibata T. Cell chirality induces collective cell migration in epithelial sheets. Phys. Rev. Lett. 115, 188102 (2015). 26565500 10.1103/PhysRevLett.115.188102

[b35] Taniguchi K. *et al.* Chirality in planar cell shape contributes to left-right asymmetric epithelial morphogenesis. Science 333, 339–341 (2011). 21764746 10.1126/science.1200940

[b36] Hozumi S. *et al.* An unconventional myosin in *Drosophila* reverses the default handedness in visceral organs. Nature 440, 798–802 (2006). 16598258 10.1038/nature04625

[b37] Petzoldt A. G. *et al.* DE-Cadherin regulates unconventional Myosin ID and Myosin IC in Drosophila left-right asymmetry establishment. Development 139, 1874–1884 (2012). 22491943 10.1242/dev.047589

[b38] Levayer R. & Lecuit T. Oscillation and polarity of E-cadherin asymmetries control actomyosin flow patterns during morphogenesis. Dev. Cell 26, 162–175 (2013). 23871590 10.1016/j.devcel.2013.06.020

[b39] Pare A. C. *et al.* A positional Toll receptor code directs convergent extension in *Drosophila*. Nature 515, 523–527 (2014). 25363762 10.1038/nature13953 PMC4943584

[b40] Rosenblatt J., Raff M. C. & Cramer L. P. An epithelial cell destined for apoptosis signals its neighbors to extrude it by an actin- and myosin-dependent mechanism. Curr. Biol. 11, 1847–1857 (2001). 11728307 10.1016/s0960-9822(01)00587-5

[b41] Toyama Y., Peralta X. G., Wells A. R., Kiehart D. P. & Edwards G. S. Apoptotic force and tissue dynamics during *Drosophila* embryogenesis. Science 321, 1683–1686 (2008). 18802000 10.1126/science.1157052 PMC2757114

[b42] Legoff L., Rouault H. & Lecuit T. A global pattern of mechanical stress polarizes cell divisions and cell shape in the growing *Drosophila* wing disc. Development 140, 4051–4059 (2013). 24046320 10.1242/dev.090878

[b43] Monier B. *et al.* Apico-basal forces exerted by apoptotic cells drive epithelium folding. Nature 518, 245–248 (2015). 25607361 10.1038/nature14152

[b44] Haas P. & Gilmour D. Chemokine signaling mediates self-organizing tissue migration in the zebrafish lateral line. Dev. Cell 10, 673–680 (2006). 16678780 10.1016/j.devcel.2006.02.019

[b45] Poukkula M., Cliffe A., Changede R. & Rorth P. Cell behaviors regulated by guidance cues in collective migration of border cells. J. Cell Biol. 192, 513–524 (2011). 21300853 10.1083/jcb.201010003 PMC3101089

[b46] Cai D. *et al.* Mechanical feedback through E-cadherin promotes direction sensing during collective cell migration. Cell 157, 1146–1159 (2014). 24855950 10.1016/j.cell.2014.03.045 PMC4118667

[b47] Kiehart D. P. Wound healing: the power of the purse string. Curr. Biol. 9, R602–R605 (1999). 10469588 10.1016/s0960-9822(99)80384-4

[b48] Fraichard S. *et al.* Tenectin is a novel alphaPS2betaPS integrin ligand required for wing morphogenesis and male genital looping in *Drosophila*. Dev. Biol. 340, 504–517 (2010). 20152825 10.1016/j.ydbio.2010.02.008 PMC2854234

[b49] Quinones-Coello A. T. *et al.* Exploring strategies for protein trapping in *Drosophila*. Genetics 175, 1089–1104 (2007). 17179094 10.1534/genetics.106.065995 PMC1840052

[b50] Morin X., Daneman R., Zavortink M. & Chia W. A protein trap strategy to detect GFP-tagged proteins expressed from their endogenous loci in *Drosophila*. Proc. Natl Acad. Sci. USA 98, 15050–15055 (2001). 11742088 10.1073/pnas.261408198 PMC64981

[b51] de Navas L., Foronda D., Suzanne M. & Sanchez-Herrero E. A simple and efficient method to identify replacements of P-lacZ by P-Gal4 lines allows obtaining Gal4 insertions in the bithorax complex of *Drosophila*. Mech. Dev. 123, 860–867 (2006). 16971094 10.1016/j.mod.2006.07.010

[b52] Koto A., Kuranaga E. & Miura M. Temporal regulation of *Drosophila* IAP1 determines caspase functions in sensory organ development. J. Cell Biol. 187, 219–231 (2009). 19822670 10.1083/jcb.200905110 PMC2768825

