# Supplementary material for: Left–right asymmetric cell intercalation drives directional collective cell movement in epithelial morphogenesis
Source: Nat Commun. 2015 Dec 10;6:10074. doi: 10.1038/ncomms10074 (PMC4682055; doi:10.1038/ncomms10074)
Supplement: Supplementary Information — Supplementary Figures 1-9, Supplementary Notes 1-4 and Supplementary References [file ncomms10074-s1.pdf]

Supplementary Figures

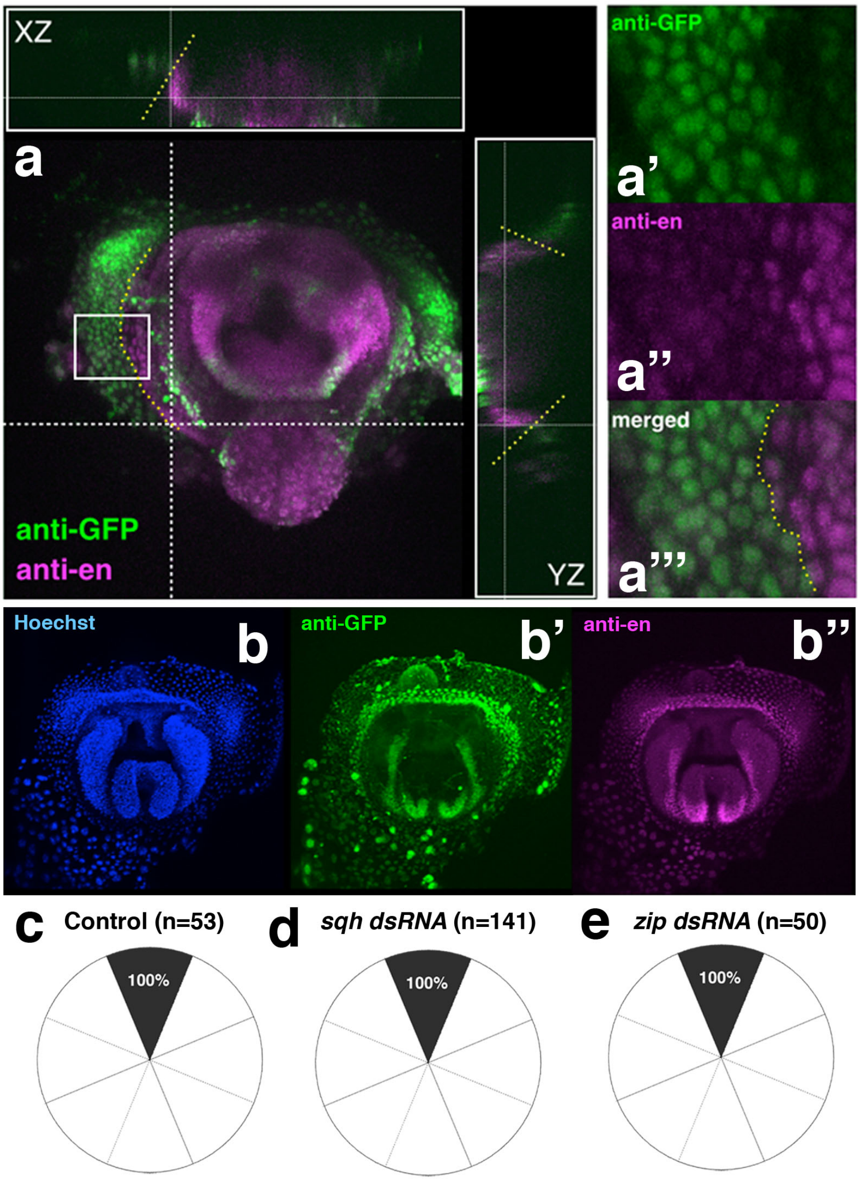

Supplementary Figure 1: The pattern of *AbdB-GAL4^{LDN}*-driven expression does not overlap that of *en-GAL4*. (a) ECFP driven by *AbdB-GAL4^{LDN}* did not overlap the anti-engrailed (en) antibody-positive region. White-boxed region was magnified in a'-a'''. (b) GFP driven by *en-GAL4* overlapped the anti-en antibody-positive region. Blue: Hoechst 33342 (b), Green: anti-GFP (b') Magenta: anti-en (b'''). (c-e) Rose diagram indicating the frequency of genitalia orientation defects at the adult stage. All of the control flies (c), *sqh* dsRNA (d) and *zip* dsRNA showed full rotation with *en-GAL4* driver. Fly genotypes: *AbdB-GAL4^{LDN}/UAS-H2B::ECFP* (a) and *en-GAL4/UAS-H2B::ECFP* (b). *en-GAL4 UAS-H2B::ECFP/UAS-LacZ dsRNA; UAS-Dcr2/+* (c), *en-GAL4 UAS-H2B::ECFP/+; UAS-Dcr2/UAS-sqh dsRNA* (d), *en-GAL4 UAS-H2B::ECFP/+; UAS-Dcr2/UAS-zip dsRNA* (e).

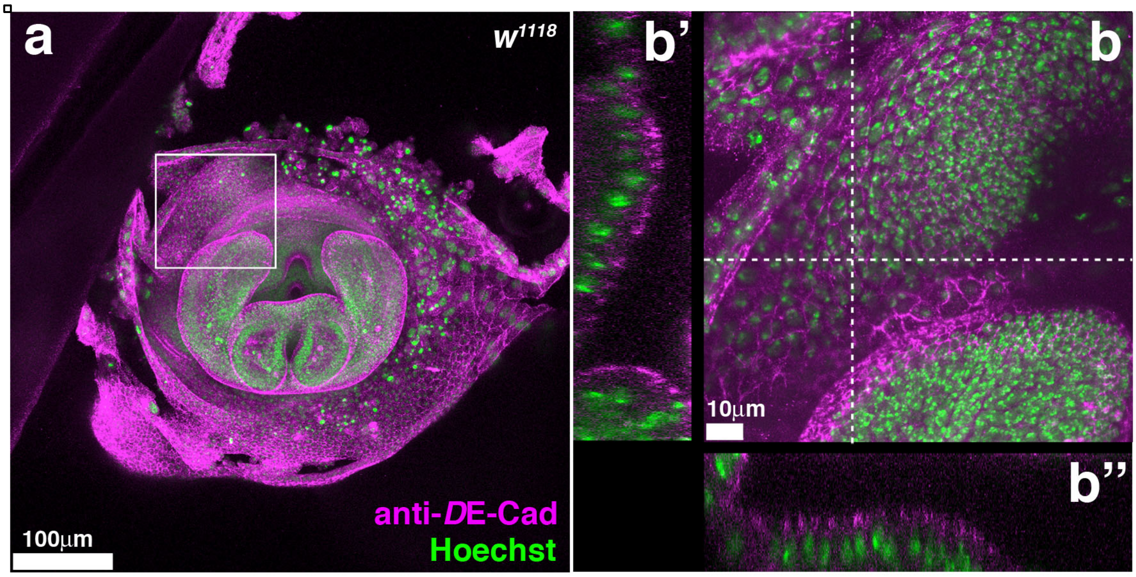

Supplementary Figure 2: Cells in A8 maintain adherence junctions with each other during rotation. Endogenous *D*E-Cad was stained using an anti-*D*E-Cad antibody (magenta). All of the nuclei were stained by Hoechst33342 (green). (b) is a magnified view of the white boxed region in (a). (b') is the YZ view of the vertical dotted line in (b), and (b'') is the XZ view of the horizontal dotted line in (b).

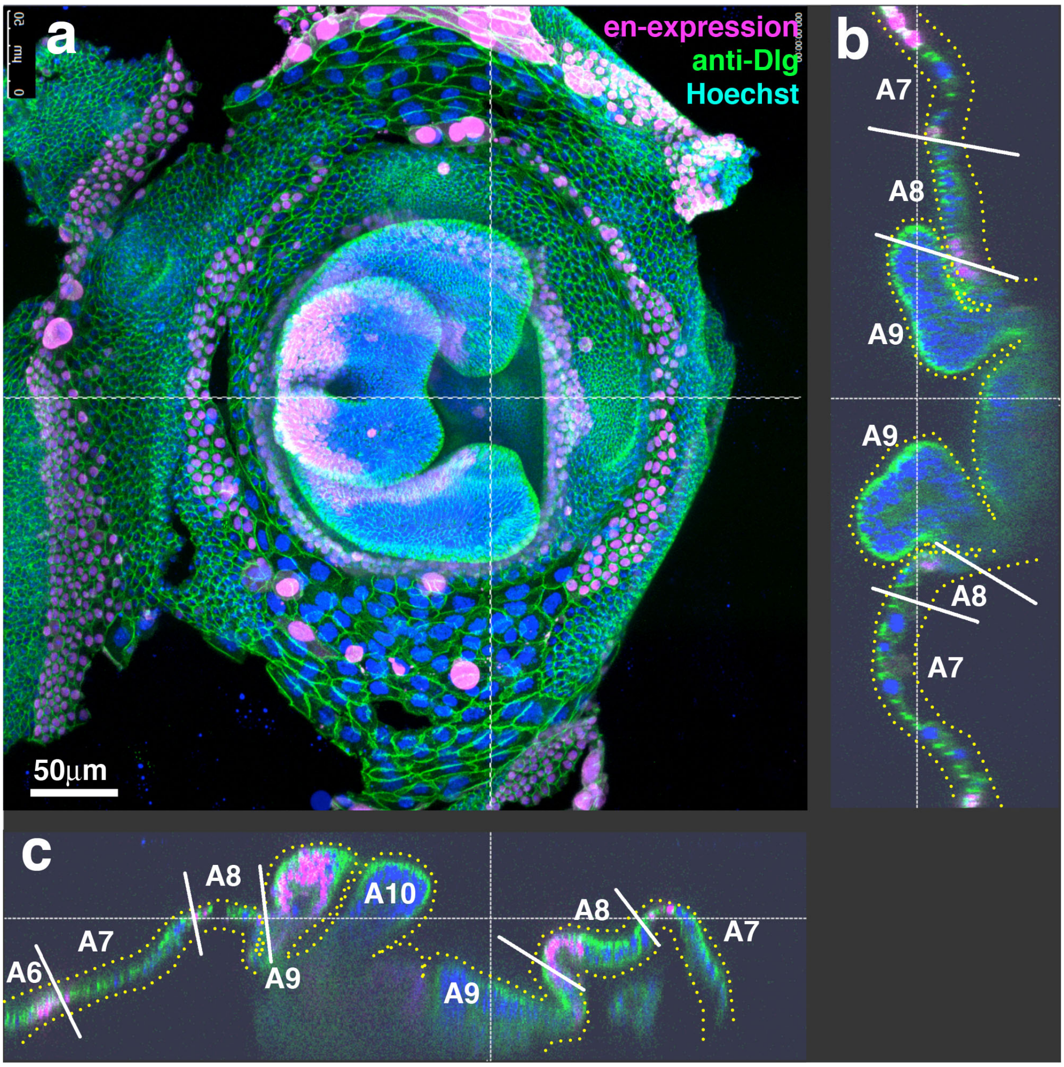

Supplementary Figure 3: The A8 tergite forms as a monolayer sheet-like disc structure that surrounds the A9 genitalia. (a-c). Endogenous Discs Large (Dlg) was stained with an anti-Dlg antibody during rotation (at 29 h APF). A fluorescent protein driven by *en-GAL4* was used as a marker for the posterior region of each segment to identify the segment borders. (b and c) Cross-sectional views of the genital disc in (a); the genital disc is indicated by a yellow dotted line. Partitions between segments are indicated by white lines. Fly genotype: *en-GAL4 UAS-H2B::ECFP/+; His2Av::mRFP/+*.

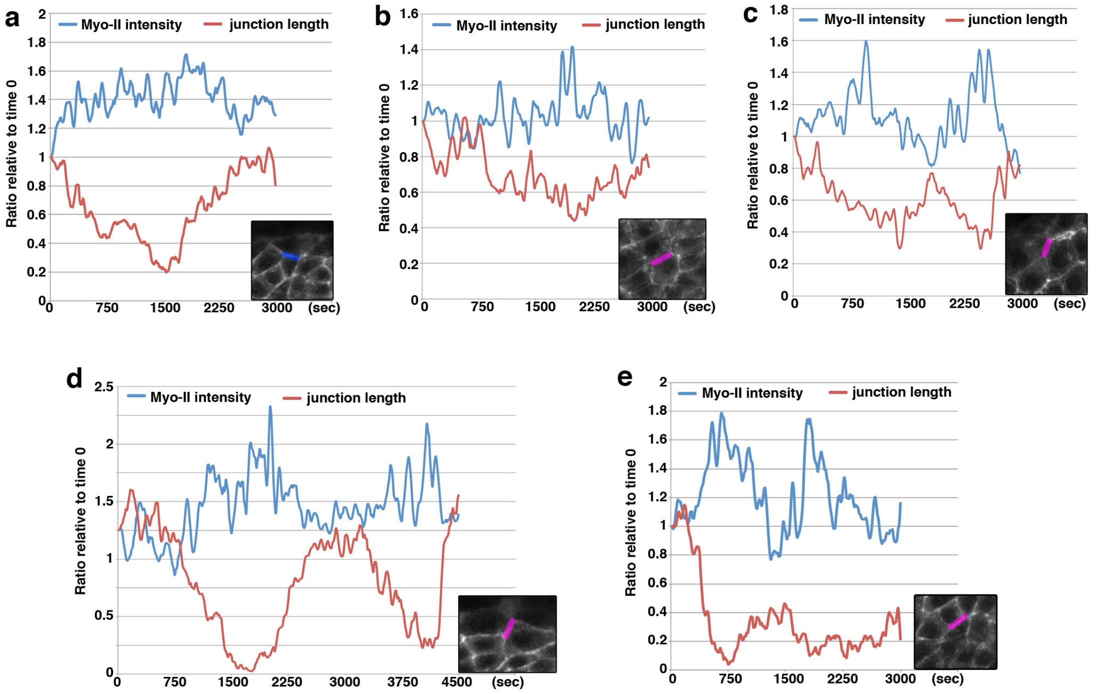

Supplementary Figure 4: Inverse relationship between Myo-II accumulation and junction shortening. Myo-II was observed as *sqh::GFP*. The intensity of *sqh::GFP* (blue lines) and junction length (red lines) over time were calculated by ImageJ at cell junctions not showing cell intercalation (a-c) and at those showing cell intercalation (d and e). Ratios relative to time zero are shown. Images at right-bottom show the junction used for each determination (*sqh::GFP*). Measurements were obtained every 15 sec.

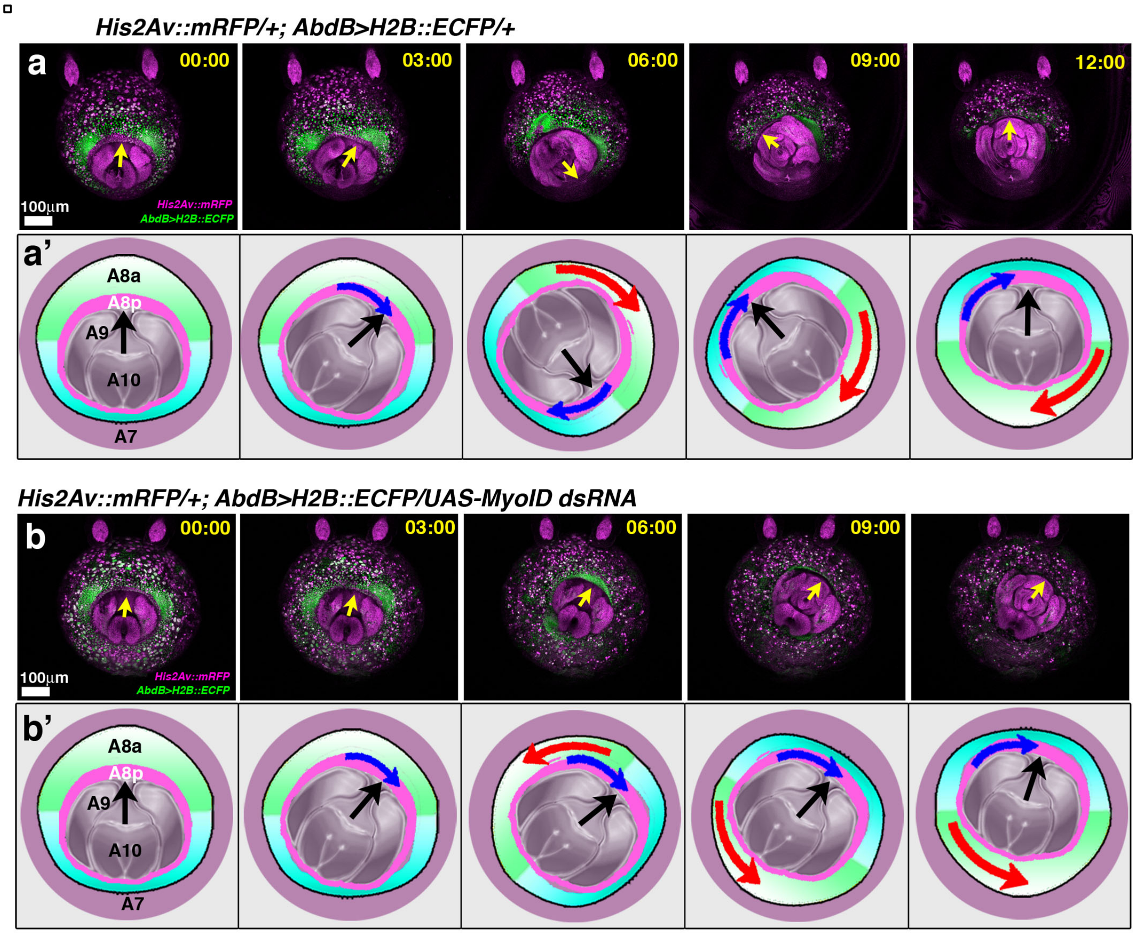

Supplementary Figure 5: Knockdown of *myoID* causes the counter-clockwise movement of A8a while A8p rotates clockwise, resulting in an apparent lack of genitalia rotation. Time-lapse series of genitalia rotation in control (a) and *myoID* knockdown (b) flies. Ventral is at the top. Green: nuclei in the anterior A8 (A8a) cells, visualized by ECFP expressed with the *AbdB-GAL4*^{LDN} driver. Magenta: all nuclei, visualized by *His2Av::mRFP*. Yellow (or black in c') arrows indicate the direction from the analia to the external genitalia. a and b are schematically drawn in a' and b'. Blue and red arrows indicate the movement of A8p and A8a, respectively. In *myoID* knockdown flies, A8a moved anti-clockwise while A8p moved in the clockwise (normal) direction, an apparent lack of genitalia rotation (b).

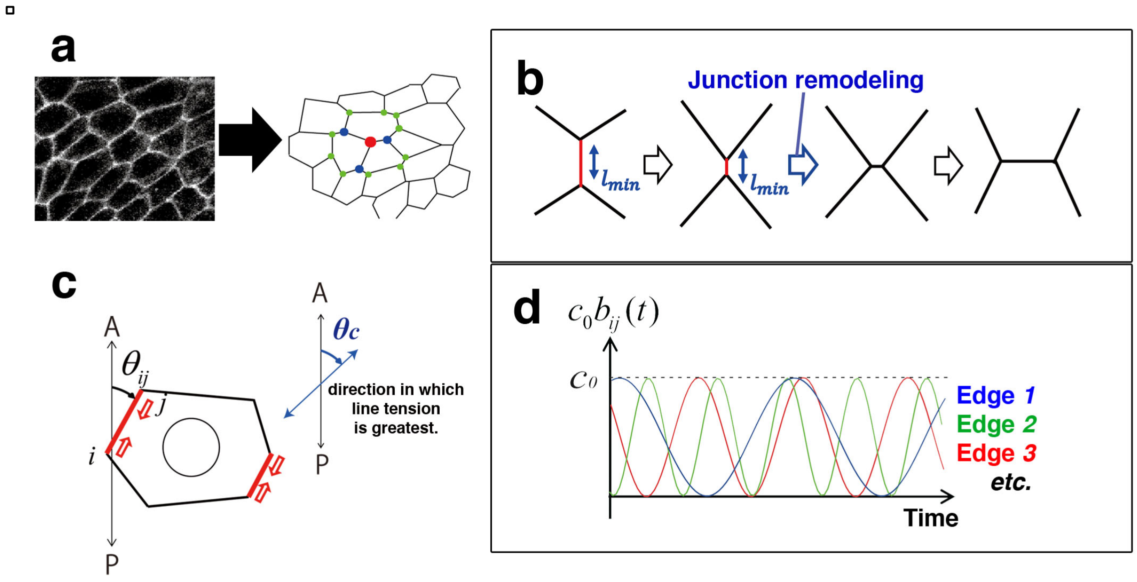

Supplementary Figure 6: Schematic representation of the vertex model (a) Cells in epithelial sheets are approximated by polygons (right), in which cell boundaries are represented by lines and the points where cell boundaries meet are represented by vertices. The green and blue points indicate the vertices that interact with the vertex indicated by the red point through cells. In addition, the vertices indicated by the blue points interact with the red one through cell boundaries. Left, fly genotype: *DE-Cad::GFP*. (b) Junction remodeling process used in our numerical simulation. When an edge length l decreased to less than l_{min} with a given length scale l_{min}, the vertex positions at the edges were rotated 90˚around the mid-point of the edge while maintaining the length l. (c) Direction dependence of line tension acting on cell boundaries. This setup represents left-right asymmetric cell polarity in our model. θ_c specifies the direction in which the line tension is greatest. (d) Schematic representation of the fluctuation in anisotropic contraction in our simulation given by eq. [4] in the Supplementary Notes.

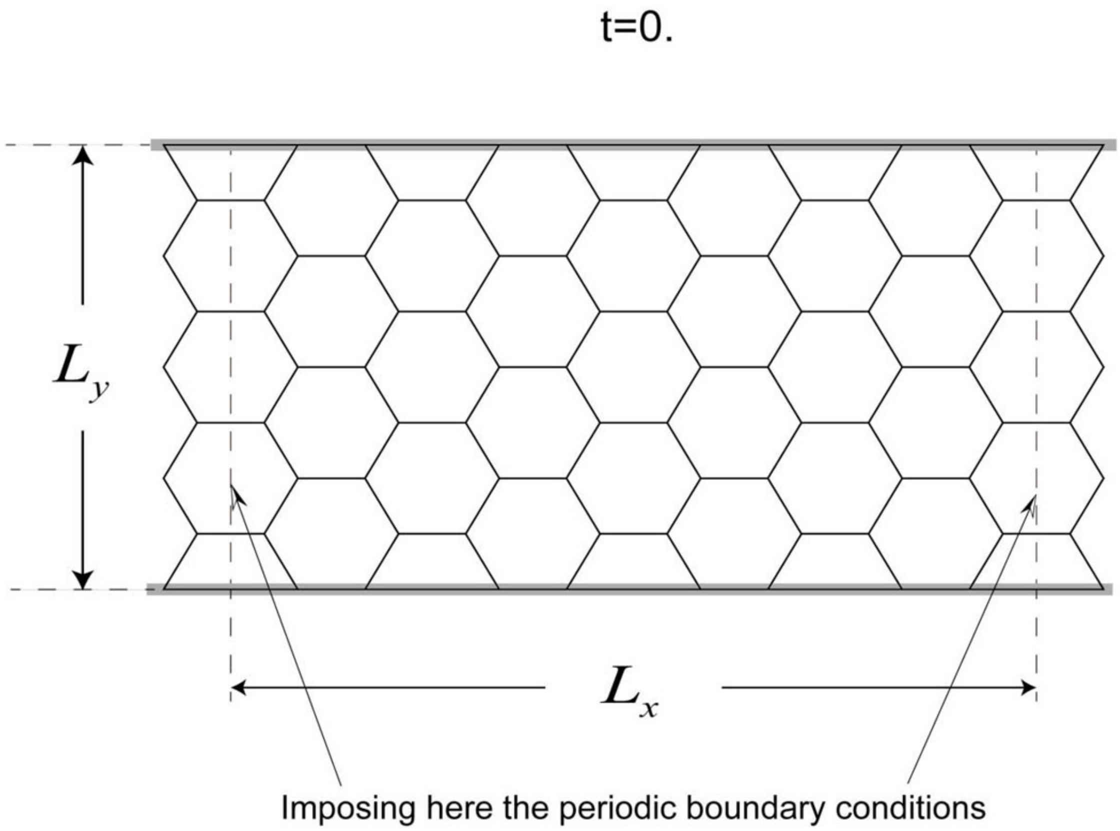

Supplementary Figure 7: Initial configuration of cells in the case of flat boundaries. The periodic boundary conditions are imposed on the boundaries indicated by the arrows. The movement of the whole epithelial sheet does not depend on the initial configuration of the cells.

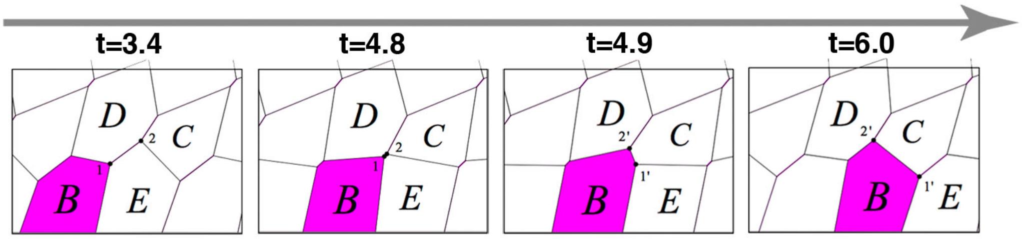

Supplementary Figure 8: Process of the continuous shear motion of epithelial cells. The four figures show enlarged snapshots around cell B in the numerical simulation given in Fig. 4a. When t=3.4, the cell boundary between cells D and E is inclined to the right with respect to the vertical (AP) axis, so that boundary has a strong contraction force and shrinks. This shrinkage of the boundary 1-2 leads to the cell configuration shown at t=4.8, at which vertices 1 and 2 are so close to each other that it undergoes junction remodeling (T1 transition). This junction remodeling deletes the boundary between cells D and E and creates a new boundary between cells B and C, cell boundary 1'-2'. The direction of cell boundary 1'-2' is inclined to the left, and this cell boundary has a relatively weak contraction force to expand. These successive dynamics of cell boundaries lead to the continuous shear movement of epithelial cells. Note that this movement is achieved only by the property of each cell to try to shrink its cell boundaries on the upper left and lower right and to expand those on the upper right and lower left.

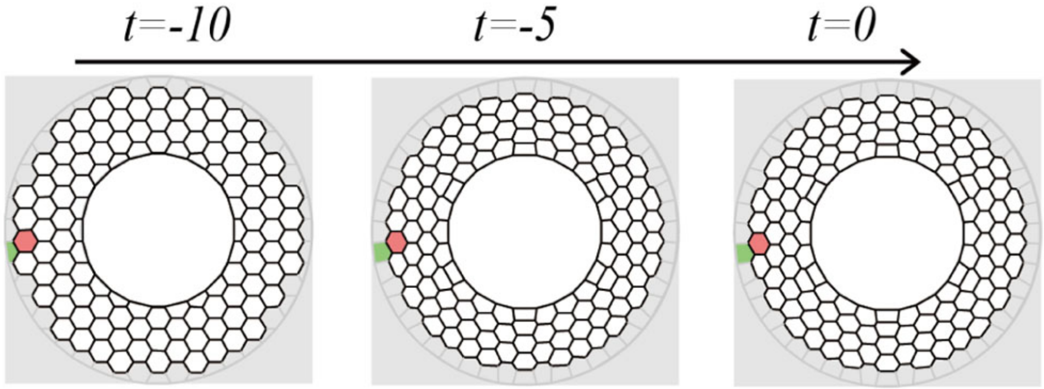

Supplementary Figure 9: Preparation of the initial configuration for the numerical simulation based on vertex dynamics associated with circular boundaries. Procedure for setting the initial condition in our numerical simulation for the 2-dimensional ring. The initial condition is given by the time $t = -10$ to $t = 0$, and obeys the equation of motion without anisotropic tension. We assumed a regular hexagonal grid of vertices at $t = -10$.

Supplementary Note 1

The vertex model: A mathematical model that describes the dynamics of epithelial sheets

To theoretically investigate the unidirectional motion of the epithelial sheet, we used a vertex model[1-5], in which important characteristic features of epithelial cells, such as PCP, cell intercalation, and their dynamics are described. We extended this model to describe the situations we are concerned with, i.e., the case in which cells in the sheet have LR asymmetric planar polarity and the epithelial sheet under consideration is confined by other types of epithelial sheets.

Supplementary Note 2 Brief review of the conventional vertex model

In the vertex model, the boundaries (interfaces) of epithelial cells are approximated by linear segments, and the cells are represented by polygons in a 2D plane (Supplementary Figure 6a). The linear segments comprising the polygons are sometimes called bonds or linear edges. The points where linear segments meet are called "vertices," and the positions and junction relationships of the vertices completely determine the cell configuration of the sheet (Supplementary Figure 6a).

The model assumes that each vertex bears forces arising from various elements in the epithelial cells, such as adherens junctions. These forces are represented by a potential U (whose explicit form is given below). The vertex also bears a frictional force that is proportional to the velocity of the vertex, which expresses the difficulty of the motion of each vertex and hence junction remodeling progression. Time evolutions of the positions of the vertices are given by

$$\mu_i \frac{d}{dt}\vec{r_i} = -\nabla_i U(\{\vec{r_j}\}, t) \quad ,\text{--[1]}$$

where $\vec{r_i} = (x_i, y_i)$ is the position vector of the i-th vertex. Equation [1] expresses the balance of the frictional force $-\mu_i \frac{d}{dt}\vec{r_i}$ and potential forces $-\nabla_i U(\{\vec{r_j}\}, t)$. Here, μ_i is the friction coefficient. In our model, μ_i is allowed to depend on the vertex index i, which is based on the assumption that the rate of change of the length of cell boundaries may depend on the cell type. The potential energy U that is derived from the forces acting on the vertices except for the friction forces is given as a function of time and the positions of all the vertices, $\{\vec{r_i}\} = \{\vec{r_1}, \vec{r_2}, \ldots, \vec{r_M}\}$, where M is the total number of vertices. The symbol ∇_i in eq. [1] means $\nabla_i = \partial / \partial \vec{r_i}$.

In our model, the potential U consists of four parts:

$$U = U_{area} + U_{perimeter} + U_{bond} + U_{boundary} .$$

Each part is explained in order below.

U_{area} is the potential energy from the cell area change, given by

$$U_{area} = \sum_{cell\ n} \frac{p_0}{2A_0}(A_n - A_0)^2 ,$$

where p_0 is the area elastic coefficient, which has the dimensions of pressure, A_n is the area of the n-th cell, and A_0 is the preferred area. This potential energy represents the pressure acting on the cell boundaries. The symbol "cell n" under the summation indicates that the index n runs from 1 to the total cell number N.

$U_{perimeter}$ is the potential energy from the cell perimeter change, given by

$$U_{perimeter} = \sum_{cell\ n} \frac{k_0}{2L_0}(L_n - L_0)^2 , \quad \text{--[2]}$$

where k_0 is the positive coefficient, L_n is the perimeter of the n-th cell, and L_0 is the preferred perimeter. This term reflects the fact that the apical area of epithelial cells is surrounded by actomyosin cables at adherens junctions, and bears a tensile force from the cables. This force tends to keep the cell shape round when L_0 is smaller than $\sqrt{4\pi A_0}$.

U_{bond} is the potential energy from the change in edge length, given by

$$U_{bond} = \sum_{\langle ij \rangle} \gamma_{ij}(t) l_{ij} , \text{--[3]}$$

where l_{ij} is the length of the edge connecting the i- and j-th vertices, given by $l_{ij} = \sqrt{(x_i - x_j)^2 + (y_i - y_j)^2}$. The index $\langle ij \rangle$ under the summation indicates that the

summation is taken over all the edges. The quantity γ_{ij} represents the line tension acting on the cell boundary ij, resulting from the contraction force from the actomyosin network at the adherens junctions.

The vertex model allows a junction remodeling process whose rules are given as [4]: When an edge length l_{ij} becomes shorter than a given length $l_{\min}$, the edge is rotated by 90 degrees about the midpoint of the edge, and the connection rule of the vertices related to the edge are rearranged such that the T1 transition is achieved (Supplementary Figure 6b).

Supplementary Note 3 Our model

Our observations showed that Myo-II levels depend on the direction of the cell boundaries. This observation implies that the line tensions acting on the cell boundaries may also depend on the line's direction. To incorporate this implication into the model, we replaced the line tension γ_{ij} in the force balance equation (eq. [1] after the derivative ∇_i is performed) with its LR asymmetric form (chirality)

$$\gamma_{ij}^{(chirality)}(t) = c_0 b_{\mathrm{ij}}(t)\cos^2(\theta_{ij} - \theta_c), \quad \text{--[4]}$$

where θ_{ij} is the angle between the anterior-posterior (AP) axis and the direction of the cell boundary ij. (Note that the sign of θ_{ij} is defined as positive if the direction of the angle of the cell boundary from the AP axis is clockwise.) θ_c represents the direction in which the line tension is greatest (Supplementary Figure 6c). Our observations allowed us to set $\theta_c = \pi/4$ (Figure 4h and 4i). c_0 is a constant representing the strength of the polarity.

It should be noted that since our model can explicitly depend on time through $\gamma_{ij}^{(chilarity)}$, the total potential energy U does not necessarily decrease with time. Our setup that γ_{ij} is replaced by $\gamma_{ij}^{(chilarity)}$ in eq. [1] guarantees that $\gamma_{ij}^{(chilarity)}$ is the force acting on the cell boundary ij.

The coefficient $b_{ij}(t)$ in eq. [4] represents the explicit time dependence of γ_{ij}. Our experiments showed that the length of cell boundaries fluctuates, implying that line tensions fluctuate. Hence, we represent this fluctuation by the coefficient b_{ij} as

$$b_{ij}(t) = \frac{1+\cos(2\pi f_{ij} t + \delta_{ij})}{2}, \quad \text{--[5]}$$

with random constant frequencies $f_{ij} \in [0,1]$ and random initial phases $\delta_{\mathrm{ij}} \in [0,2\pi)$ for each cell boundary ij (Supplementary Figure 6d). Note that when $f_{ij} = 0$, γ_{ij} does not fluctuate over time.

In our model, we must explicitly consider the boundaries of the epithelial sheet, because the tergite A8 we are concerned with is flanked by other segments, A7 and A9, whose properties are different from that of A8. The A7 segment does not move during genitalia rotation, so we consider A7 as a fixed object in space. Since A9 moves during the rotation, we regard the whole A9 as a solid disk that can move with a frictional force. Since the cells at the edges of A8 attach to these objects, the movement of these cells is constrained. This effect is expressed in terms of the potential $U_{boundary}{}^{(i)}$, which holds the i-th vertex on the edge of the sheet. The explicit form of $U_{boundary}{}^{(i)}$ reflects the shape of the sheet boundaries, so after the boundary shape is given, we can provide the explicit forms (see next section). The total potential representing the constraint of movement is formally written as

$$U_{boundary} = \sum_{vertex\ (i)\ on\ the\ sheet\ boundaries} U_{boundary}{}^{(i)}$$

where the index i includes all of the vertex indices on the boundaries of the sheet.

Supplementary Note 4 Two boundary cases in our model

(1) Flat boundary case

To examine whether or not the whole epithelial sheet undergoes unidirectional motion, we consider the simplest case, a flat system shown in Figure 5a,b and Supplementary Figure 7. In this system, the epithelial sheet is confined within the region $-L_y/2 < y < L_y/2$ by two

parallel plates, and periodic boundary conditions are imposed at the boundaries ($x = \pm L_x/2$). This situation is equivalent to one in which the epithelial sheet forms a closed band with width L_y and fixed diameter L_y/π. The boundary potential $U_{boundary}{}^{(i)}$ is given as

$$U_{boundary}{}^{(i)} = \frac{1}{2}k_{\text{constraint}}(y_i - \frac{L_y}{2})^2$$

when the i-th vertex is on the upper boundary, and

$$U_{boundary}{}^{(i)} = \frac{1}{2}k_{\text{constraint}}(y_i + \frac{L_y}{2})^2$$

when the i-th vertex is on the lower boundary. Here, $k_{\text{constraint}}$ is a large positive constant. The initial configuration of the cells is given as in Supplementary Figure 7. The AP axis is assumed to be the y-axis. The parameters used here are $L_x = \sqrt{3}$, $L_y = 1$, $A_0 = L_x L_y / N = 0.048$, $L_0 = 2\pi\sqrt{A_0/\pi} = 0.78$, $p_0 = 0.2$, $k_0 = 0.78$, $c_0 = 0.35$, $k_{\text{constraint}} = 100.0$, $l_{\min} = 0.01$ and $N = 36$. For the numerical calculation, we applied a simple Euler method with a time increment $\Delta t = 0.001$.

We set the value of μ_i as $\mu_i = 1.0$ if the i-th vertex is not on the sheet boundaries $y = \pm L_y/2$. When the vertex is on the upper boundary, the friction coefficient is given as $\mu_i = \mu_{\text{upper}}$, and when the vertex is on the lower one, $\mu_i = \mu_{\text{lower}}$. In general, μ_{upper} and μ_{lower} are different, because the epithelial sheet is flanked by different segments, A7 and A8p. We examine the following two cases.

(i) When μ_{upper} and μ_{lower} are equal ($\mu_{\text{upper}} = \mu_{\text{lower}} = 1.0$)

According to the symmetry arguments, if there is no inhomogeneity along the y-axis, the whole epithelial sheet does not move in the x-direction, even when any time sequences of line tensions are allowed. As this argument states, in the case of $\mu_{\text{upper}} = \mu_{\text{lower}}$, each cell can move in the x-axis, but the whole sheet does not move (Figure 5a and Supplementary Movie 6).

(ii) When μ_{upper} is greater than μ_{lower} ($\mu_{\text{upper}} = 100.0$, $\mu_{\text{lower}} = 1.0$)

In this case, inhomogeneity along the AP axis exists, so the whole set of epithelial cells, i.e., the center of mass of the sheet, can move in the x-direction (Figure 5b and Supplementary Movie 7).

(2) Circular boundary case

To compare the results of the numerical simulations with the experimental observations, we examine circular boundaries as shown in Figure 5c, where we call the outer boundary the outer ring, and the inner boundary the inner ring. The boundary potential energy $U_{boundary}{}^{(i)}$ is given as

$$U_{boundary}{}^{(i)} = \frac{1}{2} k_{\text{constraint}} (\sqrt{x_i^2 + y_i^2} \quad R_{A8})^2$$

when the i-th vertex is on the outer ring, and

$$U_{boundary}{}^{(i)} = \frac{1}{2} k_{\text{constraint}} (\sqrt{x_i^2 + y_i^2} \quad R_{A9})^2$$

when the vertex is on the inner ring. Here, R_{A8} and R_{A9} are the radius of the outer ring and of the inner ring, respectively. The initial conditions are given by the configuration shown in Supplementary Figure 9, right. (This configuration is prepared by the numerical time evolution from $t = -10$ to $t = 0$ obeying the equation of motion for $c_0 = 0$. At $t = -10$, the vertices are set as a regular hexagonal grid (Supplementary Figure 9 left).) The AP axis is assumed to be the direction specified by the ray from the center of the outer ring to the center of mass of the n-th cell. The centers of both the outer and inner rings are set to be the origin of the x-y plane. The parameters used here are $R_{A8} = 1.0$, $R_{A9} = 0.5$, $k_{\text{constraint}} = 100.0$, $A_0 = \frac{\pi R_{A8}{}^2 - \pi R_{A9}{}^2}{N} \sim 0.014$, $L_0 = 0.8 \times \sqrt{8\sqrt{3} A_0} \sim 0.35$, $p_0 = 10.0$, $k_0 = 1.0$, $c_0 = 0.6$, $l_{min} = 0.005$, and $N = 168$. The friction coefficient μ_i is given as $\mu_i = 1.0$ when the vertex i is not on the circular boundaries. If the vertex is on the outer ring, $\mu_i = \mu_{A7} = 100.0$, and if the vertex is on the inner ring, $\mu_i = \mu_{A9} = 1.0$. Fluctuation is included as $f_{ij} \in [0,1]$

and $\delta_{ij} \in [0,2\pi)$.

We performed the numerical simulation with this setting, and found that the cell population can rotate continuously (Figure 5c and Supplementary Movie 8).

Supplementary References

1 Farhadifar, R., Roper, J. C., Aigouy, B., Eaton, S. & Julicher, F. The influence of cell mechanics, cell-cell interactions, and proliferation on epithelial packing. *Curr Biol* **17**, 2095-2104, (2007).

2 Rauzi, M., Verant, P., Lecuit, T. & Lenne, P. F. Nature and anisotropy of cortical forces orienting Drosophila tissue morphogenesis. *Nat Cell Biol* **10**, 1401-1410, (2008).

3 Weliky, M. & Oster, G. The mechanical basis of cell rearrangement. I. Epithelial morphogenesis during Fundulus epiboly. *Development* **109**, 373-386 (1990).

4 Nagai, T. & Honda, H. A dynamic cell model for the formation of epithelial tissue. *Philos. Mag.* **B81**, 699-719 (2001).

5 Staple, D. B. *et al.* Mechanics and remodelling of cell packings in epithelia. *Eur Phys J E Soft Matter* **33**, 117-127, (2010).
